# Phase‐encoded fMRI tracks down brainstorms of natural language processing with subsecond precision

**DOI:** 10.1002/hbm.26617

**Published:** 2024-02-09

**Authors:** Victoria Lai Cheng Lei, Teng Ieng Leong, Cheok Teng Leong, Lili Liu, Chi Un Choi, Martin I. Sereno, Defeng Li, Ruey‐Song Huang

**Affiliations:** ^1^ Centre for Cognitive and Brain Sciences University of Macau Taipa China; ^2^ Faculty of Arts and Humanities University of Macau Taipa China; ^3^ Faculty of Science and Technology University of Macau Taipa China; ^4^ Department of Psychology San Diego State University San Diego California USA

**Keywords:** brainstorms, dual‐stream models, hemodynamic traveling waves, information flows, logistics models

## Abstract

Natural language processing unfolds information overtime as spatially separated, multimodal, and interconnected neural processes. Existing noninvasive subtraction‐based neuroimaging techniques cannot simultaneously achieve the spatial and temporal resolutions required to visualize ongoing information flows across the whole brain. Here we have developed rapid phase‐encoded designs to fully exploit the temporal information latent in functional magnetic resonance imaging data, as well as overcoming scanner noise and head‐motion challenges during overt language tasks. We captured real‐time information flows as coherent hemodynamic waves traveling over the cortical surface during listening, reading aloud, reciting, and oral cross‐language interpreting tasks. We were able to observe the timing, location, direction, and surge of traveling waves in all language tasks, which were visualized as “brainstorms” on brain “weather” maps. The paths of hemodynamic traveling waves provide direct evidence for dual‐stream models of the visual and auditory systems as well as logistics models for crossmodal and cross‐language processing. Specifically, we have tracked down the step‐by‐step processing of written or spoken sentences first being received and processed by the visual or auditory streams, carried across language and domain‐general cognitive regions, and finally delivered as overt speeches monitored through the auditory cortex, which gives a complete picture of information flows across the brain during natural language functioning.

**Practitioner Points:**

- Phase‐encoded fMRI enables simultaneous imaging of high spatial and temporal resolution, capturing continuous spatiotemporal dynamics of the entire brain during real‐time overt natural language tasks.
- Spatiotemporal traveling wave patterns provide direct evidence for constructing comprehensive and explicit models of human information processing.
- This study unlocks the potential of applying rapid phase‐encoded fMRI to indirectly track the underlying neural information flows of sequential sensory, motor, and high‐order cognitive processes.

## Graphical abstract

Phase‐encoded fMRI captures the step‐by‐step spatiotemporal brain dynamics of cognitive processes. When a sentence is heard, language information is carried across the auditory cortex in multiple streams of traveling waves. This provides direct evidence for and supplement the dual‐stream model of speech processing.

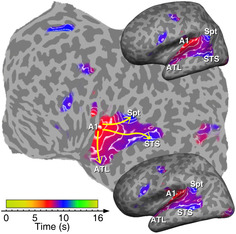

## 1 INTRODUCTION

Language plays a central role in human information processing. Existing cognitive models have depicted the flow of information from perception to response, with various processing stages in between (Atkinson & Shiffrin, 1968; Engelkamp, 1998; Miller, 1956; Wickens, 1992). Neural models of language processes suggest that the processing of linguistic information involves a series of steps associated with activations across the brain (Hagoort & Levelt, 2009; Indefrey & Levelt, 2004; Levelt, 1989; Levelt et al., 1999) via certain processing pathways (Coltheart et al., 2001; Hickok & Poeppel, 2007; Rauschecker & Scott, 2009). One important issue is to determine which processes operate sequentially and which in parallel (Proctor & Vu, 2012). Models describing oral interpreting explicitly suggest there are overlaps between various processes during this complex language task (Gile, 1995; Paradis, 1994). Therefore, mapping out the whole‐brain spatiotemporal dynamics of natural language processing in real time holds the key to the understanding of language and cognitive functioning. Demonstrating how different brain regions interact with each other during language perception and production has been identified as one of the greatest challenges (Price, 2012).

Both spatial and temporal characterizations of brain activities are important for revealing the neural mechanisms of language perception and production and their interaction with domain‐general cognitive functions (Fedorenko & Thompson‐Schill, 2014). Invasive procedures like electrocorticography or implanted electrodes have recorded the neural activities of language processing with high spatial and temporal resolution from local regions, but cannot record whole‐brain activity (Bouchard et al., 2013; Castellucci et al., 2022; Chartier et al., 2018; Mesgarani et al., 2014; Sahin et al., 2009). Existing noninvasive neuroimaging techniques such as magnetoencephalography and functional magnetic resonance imaging (fMRI) can only achieve either millisecond or millimeter precision, but not both (Indefrey & Levelt, 2004; Price, 2010, 2012). Due to its lower temporal resolution and the technical challenges, including scanner noise and speaking induced‐head motion artifacts (Jolly et al., 2020), fMRI has been somewhat overlooked for capturing the continuous processing of natural language comprehension and production at the second‐to‐second level. Sparse‐sampling fMRI can localize brain activations associated with overt vocal responses during silent delays between acquisitions (Perrachione & Ghosh, 2013), but cannot reveal the temporal order of the ongoing brain activities within and beyond these regions.

While recent fMRI studies have demonstrated sufficient temporal resolution for revealing spontaneous neural progression waves at resting states (Bolt et al., 2022; Gu et al., 2021; Pines et al., 2023; Raut et al., 2021), how event‐related language activations propagate through the brain remain obscure. Several studies have used time‐resolved fMRI to determine the activation order of distinct brain regions during language perception and production tasks or bilingual control tasks (Humphries et al., 2007; Janssen & Mendieta, 2020; Reverberi et al., 2015; Sigman et al., 2007). However, how information flows continuously from region to region across the language network remains less clear. Also, there is still a lack of direct evidence for the understanding of auditory feedback at the speech production stage. In this study, we used real‐time phase‐encoded fMRI to capture and unravel the spatiotemporal traveling waves of blood oxygen level dependent (BOLD) signals across the cortical surface during naturalistic language tasks, which involved overt speech. Phase‐encoded designs have long been developed and used to map the topological organization of human occipital, parietal, frontal, auditory, and sensorimotor cortices (Engel et al., 1994; Huang et al., 2012, 2017; Huang & Sereno, 2013; Sereno et al., 1995, 2001, 2022; Sereno & Huang, 2006; Sood & Sereno, 2016). In a typical phase‐encoded retinotopic mapping experiment, for example, a wedge containing flickering checkerboard pattern or live‐feed videos slowly revolves around a central fixation for a period of 64 s (Huang & Sereno, 2013; Sereno et al., 1995; Sereno & Huang, 2006; Sood & Sereno, 2016). Cortical patches representing different polar angles are thus stimulated at different phases (time delays). Fourier analysis of resulting fMRI time series reveals periodic brain activities and their phases at the stimulus frequency (e.g., eight cycles per scan), often visualized as surface waves traveling across polar‐angle representations within each retinotopic area (Sereno et al., 1995, 2001; Sereno & Huang, 2006; Sood & Sereno, 2016). The very slow rate of stimulus progression was initially chosen to respect the slow hemodynamic response. However, it was yet to be determined whether phase‐encoded designs could be used to capture the faster brain dynamics during real‐time language processing.

A recent phase‐encoded fMRI study demonstrates that the activation sequence of sensorimotor transformations during a periodic reach‐to‐eat task can be decoded within a period as short as 16 s (Chen et al., 2019). Inspired by this recent success and slow event‐related designs consisting of speech perception and production phases in previous studies (Buchsbaum et al., 2001; Hickok et al., 2003), we conducted an fMRI experiment with a series of rapid phase‐encoded tasks involving unimodal (reading or listening in Chinese [native language, L1] or English [second language, L2]), multimodal (reading aloud or shadowing in L1 or L2), crossmodal (reading or listening and then reciting in L1 or L2), and cross‐language (crossmodal L1‐to‐L2 or L2‐to‐L1) processing of natural sentences. The phase‐encoded tasks involved periodic presentation of language stimuli and subjects were required to respond to these stimuli differently, depending on task requirements. For example, in a crossmodal task, subjects were required to read and memorize a sentence for 5 s (Phase 1), recite it in the next 5 s (Phase 2), and then rest for 6 s (Phase R) in each 16‐s cycle. This phase‐encoded task sequence was repeated periodically within a single scan. From language perception to resting phases, the crossmodal task involved a sequence of cognitive processes leading to periodic brain activations with different phases within a cycle. We aimed to track down the timing, location, direction, and surge of periodic BOLD waves as spatiotemporal “brainstorm” patterns during unimodal, multimodal, crossmodal, and cross‐language tasks.

## 2 MATERIALS AND METHODS

### 2.1 Participants

Twenty‐one bilingual students (15 females; 6 males) between 20 and 42 years old (Mean: 23.5; SD: 4.9) participated in this study. All of them were native Chinese speakers with English as their second language, and they started learning English between the age of 3 and 13 (Mean: 7.5; SD: 2.6). Their levels of English proficiency were evaluated based on their recent results in standardized English language tests (IELTS 6.5+ or equivalent). All of them had normal or corrected‐to‐normal vision and no history of neurological impairment. All subjects gave written informed consent according to experimental protocols approved by the Ethics Committee of the University of Macau.

### 2.2 Experimental setup

To prevent head motion during tasks involving speaking, we made “The Phantom of the Opera” style masks molded to individual faces using thermoplastic sheets (1.6 mm H‐board, Sun Medical Products Co., Ltd.). Each subject participated in a brief training session in an MRI simulator (Shenzhen Sinorad Medical Electronics Co., Ltd.) equipped with a motion sensor (MoTrak, Psychology Software Tools, Inc.). The subject would wear a thermoplastic mask and put on a pair of headphones, and then practice keeping their head still while speaking in the MRI simulator. The head motion was monitored in real time by a sensor attached to the subject's forehead. Auditory feedback (“ding” sounds) would be heard via the headphones whenever the head motion exceeded a threshold (1 mm in translation or 1° in rotation).

**FIGURE 1:**
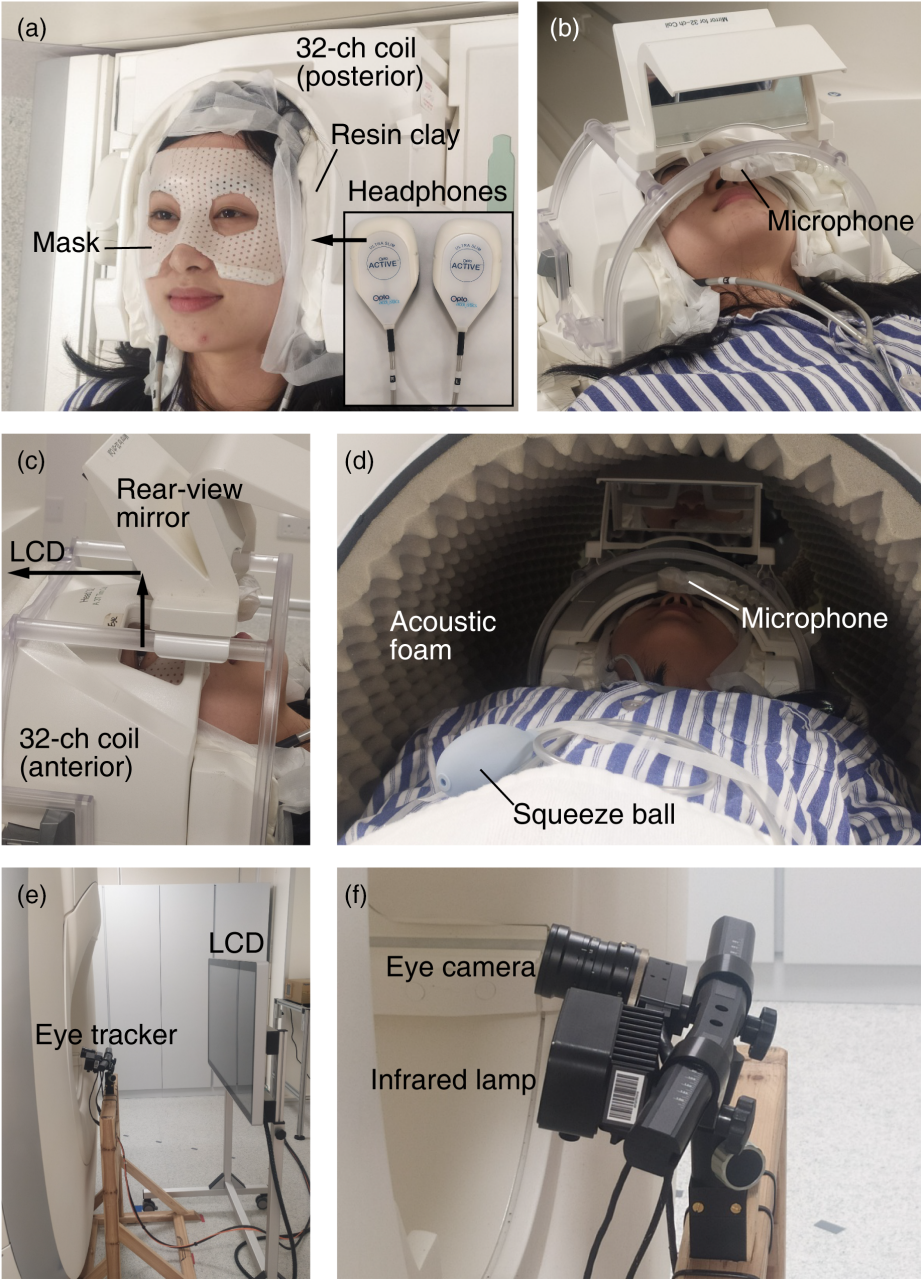
Experimental setup. (a) Subject wearing a “The Phantom of the Opera” style mask and a pair of noise‐canceling headphones, with resin clay filled around the head and headphones to reduce head motion and scanner noise. (b) A microphone in front of the subject's mouth. (c) A rear‐view mirror mounted above the 32‐channel head coil. (d) Acoustic foams placed inside the bore for scanner noise reduction. (e) MR‐compatible LCD monitor and eye tracker behind the scanner. (f) A close‐up view of the eye tracker.

Before each fMRI session, the subject would wear a thermoplastic mask and earplugs, and put on a pair of MR‐compatible noise‐canceling headphones (OptoActive II, OptoAcoustics Ltd.; Figure 1). The subject would then lie supine with the head and headphones surrounded by deformable resin clay and silicone gel inside a head coil (Figure 1a). An MR‐compatible microphone (OptoActive II, OptoAcoustics Ltd.) was placed above the subject's mouth (Figure 1b,d), which allowed the subject to hear his or her own voice in real time via the headphones. The inner wall of scanner bore was lined with acoustic foams to further reduce gradient noise (Figure 1d). A rear‐view mirror was mounted on top of the head coil, allowing the subject to view visual stimuli on an MR‐compatible 40″ LCD monitor (InroomViewingDevice, NordicNeuroLab AS) located 60 cm from the scanner bore (Figure 1c,e). The total distance between the eyes and LCD monitor (via a mirror) was about 180 cm. The visually presented sentences subtended a maximum width of 39 cm on screen, resulting in a 12.4° horizontal field of view (6.2° eccentricity). An MR‐compatible eye tracker (Eyelink 1000 Plus, SR Research Ltd.) was mounted behind the scanner bore, capturing the subject's eye movements via the mirror (Figure 1e,f). Both the mirror and the eye tracker were calibrated immediately after the subject was moved into the scanner bore. Visual and auditory stimuli were presented using Experiment Builder (SR Research Ltd.), which awaited a trigger signal (“s”) from SyncBox (NordicNeuroLab AS) before starting each functional scan.

### 2.3 Stimuli

Chinese (L1) stimuli included a total of 128 sentences, each consisting of 14 Chinese characters (see examples in Table S1). For each sentence, a subject, verb, and an object were selected from *The Lancaster Corpus of Mandarin Chinese* (https://www.lancaster.ac.uk/fass/projects/corpus/LCMC/) to construct simple sentences in the subject‐verb‐object word order. All sentences were constructed systematically by native Chinese speakers and validated by a professor of linguistics to ensure all sentences were plausible and unambiguous. English (L2) stimuli included a total of 128 sentences, each consisting of 15–17 syllables (see examples in Table S2). For the construction of each sentence, a verb was first selected from *Longman Communication 3000*, a list of 3000 most frequently used words in spoken and written English. The verb was then searched in *Collins Dictionary English* (https://www.collinsdictionary.com/dictionary/english/corpus) for sentence examples to ensure the sentences were natural. Only sentence examples that are in the simple subject‐verb‐object structure were selected and modified to meet the syllable count requirement. Finally, the modified sentence was checked with *Longman Vocabulary Checker* (http://global.longmandictionaries.com/vocabulary_checker) to ensure all the words in the sentence were middle to high‐frequency words. All sentences were then validated by a professor of linguistics to ensure they were unambiguous and plausible.

### 2.4 Experimental design and paradigm

**FIGURE 2:**
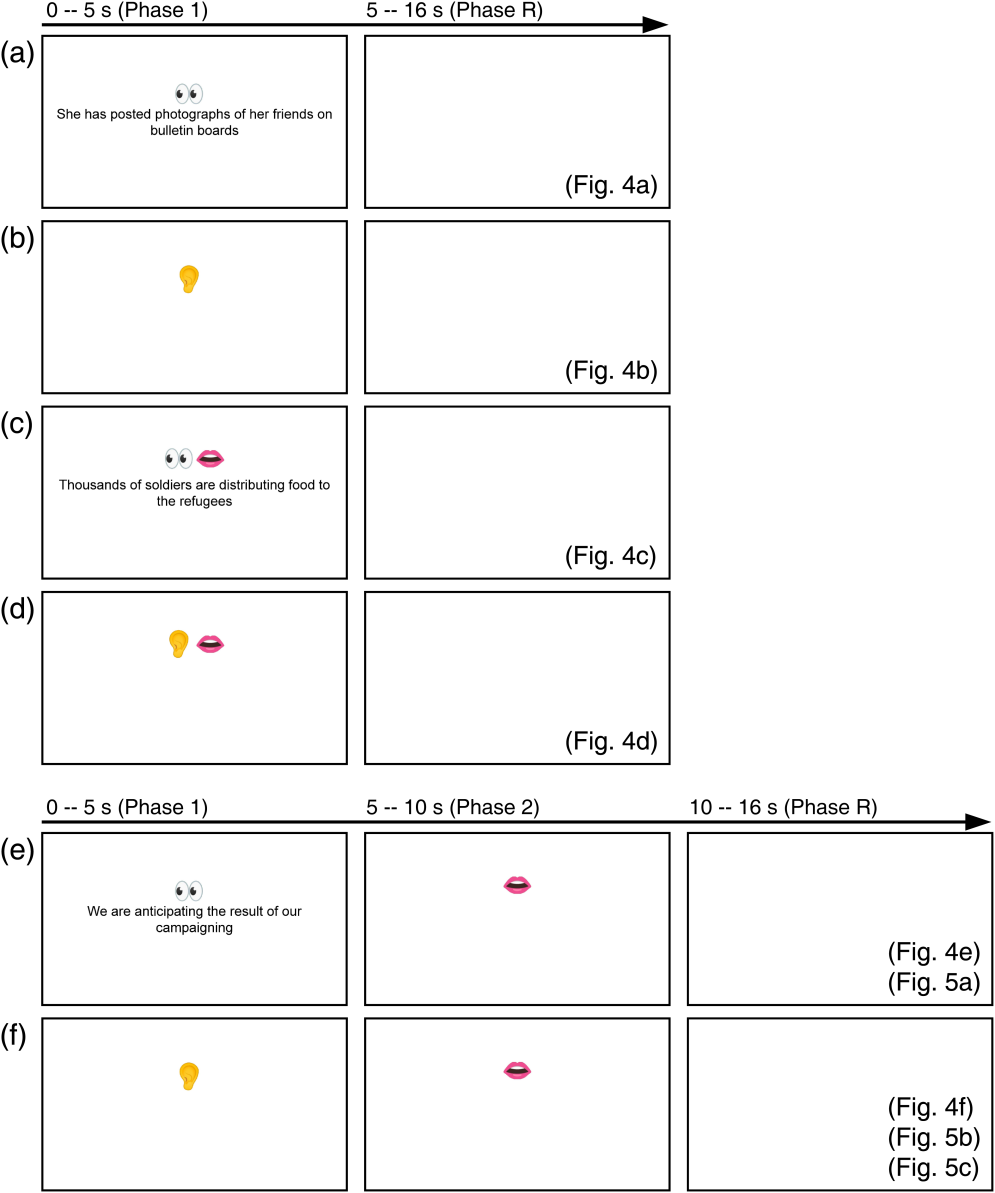
Experimental paradigms and stimuli. (a) Reading only tasks, corresponding to Figure 4a. (b) Listening only tasks, corresponding to Figure 4b. (c) Reading‐aloud tasks, corresponding to Figure 4c. (d) Shadowing tasks, corresponding to Figure 4d. (e) Reading‐memorizing‐reciting and reading‐memorizing‐translating tasks, corresponding to Figures 4e and 5a. (f) Listening‐memorizing‐reciting and listening‐memorizing‐translating tasks, corresponding to Figures 4f and 5b,c. In (a–d), visual or auditory stimuli were presented between 0 and 5 s (Phase 1), followed by a blank screen between 5 and 16 s (Phase R). In (e,f), visual or auditory stimuli were presented between 0 and 5 s (Phase 1), followed by speech output between 5 and 10 s (Phase 2) and a blank screen between 5 and 16 s (Phase R).

Each subject participated in three sessions—one Chinese language session, one English language session, and one translation (L1‐to‐L2 and L2‐to‐L1) session. The three sessions were on three different days. Each of the Chinese or English sessions consisted of twelve 256‐s phase‐encoded scans, two scans for each of the six different tasks (Figure 2):

Scan #1: Reading the visually presented sentence silently;
Scan #2: Reading aloud the sentence as soon as it is visually presented;
Scan #3: Reading silently and memorizing the visually presented sentence, then reciting it when a mouth cue appears;
Scan #4: Reading the visually presented sentence silently;
Scan #5: Reading aloud the sentence as soon as it is visually presented;
Scan #6: Reading silently and memorizing the visually presented sentence, then reciting it when a mouth cue appears;
Scan #7: Listening to the auditorily presented sentence;
Scan #8: Shadowing the auditorily presented sentence (repeating what is heard aloud right after the onset of the auditory presentation of the sentence);
Scan #9: Listening to and memorizing the auditorily presented sentence, then reciting it when a mouth cue appears;
Scan #10: Listening to the auditorily presented sentence;
Scan #11: Shadowing (repeating what is heard aloud right after the onset of the auditory presentation of the sentence);
Scan #12: Listening to and memorizing the auditorily presented sentence, then reciting it when a mouth cue appears.

Each 256‐s scan consisted of 16 randomly selected sentences, each sentence was presented visually (Scans #1 to #6) or auditorily (Scans #7 to #12) within the first 5 s of each 16‐s trial period (Figure 2). For Scans #1 to #6, each sentence was presented in black text (32 pt. Arial font), centered against a white background on an LCD monitor. For Scans #1 and #4, each sentence was presented for 5 s with a visual cue (eyes) above the sentence, followed by a blank screen for 11 s within each 16‐s period (Figure 2a). Subjects were instructed to read the sentence silently and attentively within the first 5 s (Phase 1) and then rest for 11 s (Phase R) with their eyes open. For Scans #2 and #5, each sentence was presented for 5 s with a visual cue (eyes and mouth) above the sentence followed by a blank screen for 11 s (Figure 2c). Subjects were instructed to read aloud the sentence as soon as it appeared within 5 s (Phase 1) and rest for 11 s (Phase R) with their eyes open. For Scans #3 and #6, each sentence was presented for 5 s with a visual cue (eyes) above the sentence, followed by a visual cue (mouth) for 5 s and a blank screen for 6 s (Figure 2e). Subjects were instructed to silently read and memorize the sentence within the first 5 s (Phase 1) and recite it in the next 5 s (Phase 2) when the mouth cue appeared, followed by a 6‐s rest (Phase R) with their eyes open.

In Scans #7 to #12, stimuli were presented auditorily through a pair of active noise‐canceling headphones. Subjects were instructed to listen to the speech attentively and keep their eyes open for the whole time. The speech of each sentence was synthesized using *Google Cloud Text‐to‐Speech* tool under default setting. The speech rate ranged from 2.9 to 3.1 syllables per second. For Scans #7 and #10, a speech with a visual cue (ear) was presented for 5 s (Phase 1), followed by a 11‐s blank screen (Phase R) within each 16‐s period (Figure 2b). For Scans #8 and #11, a speech and a visual cue (ear and mouth) were presented for 5 s (Phase 1), followed by a 11‐s blank period (Phase R; Figure 2d). Subjects were instructed to listen to the speech and repeat it right after it was heard. For Scans #9 and #12, a speech and a visual cue (ear) were presented for 5 s (Phase 1), followed by a visual cue (mouth) between 5 and 10 s (Phase 2) and a 6‐s blank screen between 10 and 16 s (Phase R; Figure 2f). Subjects were instructed to listen and memorize the speech during the first 5 s (Phase 1), and repeat the speech between 5 and 10 s (Phase 2).

Similar to the monolingual sessions, stimuli for the translation session were either visually or auditorily presented. Subjects would read silently or listen to a sentence in one language (Phase 1), and then orally translate it into the other language (Phase 2) when a mouth cue appeared. This was followed by a 6‐s blank screen between 10 and 16 s (Phase R; Figure 2e,f).

The translation session consisted of twelve 256‐s functional scans, two scans for each of the six different tasks:

Scan #1: Reading the visually presented Chinese sentence silently, memorizing it, then orally translating it into English (L1‐to‐L2);
Scan #2: Listening to the auditorily presented Chinese sentence, memorizing it, then orally translating it into English (L1‐to‐L2);
Scan #3: Listening to five‐digit number in Chinese, memorizing it, then orally translating it into English (L1‐to‐L2);
Scan #4: Reading the visually presented Chinese sentence silently, memorizing, then orally translating it into English (L1‐to‐L2);
Scan #5: Listening to the auditorily presented Chinese sentence, memorizing it, then orally translating it into English (L1‐to‐L2);
Scan #6: Listening to five‐digit number in Chinese, memorizing it, then orally translating it into English (L1‐to‐L2);
Scan #7: Reading the visually presented English sentence silently, memorizing it, then orally translating it into Chinese (L2‐to‐L1);
Scan #8: Listening to the auditorily presented English sentence, memorizing it, then orally translating it into Chinese (L2‐to‐L1);
Scan #9: Listening to the five‐digit number in English, memorizing it, then orally translating it into Chinese (L2‐to‐L1);
Scan #10: Reading the visually presented English sentence silently, memorizing it, then orally translating it into Chinese (L2‐to‐L1);
Scan #11: Listening to the auditorily presented English sentence, memorizing it, then orally translating it into Chinese (L2‐to‐L1);
Scan #12: Listening to the five‐digit number in English, memorizing it, then orally translating it into Chinese (L2‐to‐L1).

The presentation paradigm for the visually presented stimuli was the same as that for Scans #3 and #6 of the monolingual sessions (Figure 2e). The paradigm for presenting auditory stimuli was the same as that for Scans # 9 and #12 of the monolingual sessions (Figure 2f).

### 2.5 Behavioral recording

To ensure task compliance, speech output, and eye movements were recorded in sync with functional scans. The subject's voice was recorded into four soundtracks using the OptiMRI software (OptoAcoustics Ltd.) and analyzed using the Audacity® software (https://audacityteam.org; Figure S1). The group averages of subjects' speech onset, offset, and duration are summarized in Table 1. For tasks that involved reading, a heat map was generated using EyeLink DataViewer (SR Research Ltd.) to visualize the interest period (0–5 s within each 16‐s trial) with fixation duration and fixation count (Figure S2).

**TABLE 1:** Group average timing and standard deviation of recorded speech output. Data from the same task were averaged across 21 subjects.

Language	Task	Speech onset (s)	Speech offset (s)	Duration (s)
Chinese (L1)	Reading‐aloud (Figure 4c, left)	2.55 ± 0.15	5.58 ± 0.32	3.04 ± 0.30
	Shadowing (Figure 4d, left)	3.15 ± 0.67	7.23 ± 0.65	4.08 ± 0.24
	Reading‐memorizing‐reciting (Figure 4e, left)	7.42 ± 0.17	10.52 ± 0.53	3.16 ± 0.48
	Listening‐memorizing‐reciting (Figure 4f, left)	5.92 ± 0.15	9.07 ± 0.61	3.15 ± 0.58
English (L2)	Reading‐aloud (Figure 4c, right)	2.65 ± 0.25	6.55 ± 0.47	4.47 ± 0.37
	Shadowing (Figure 4d, right)	2.88 ± 0.29	7.51 ± 0.45	4.64 ± 0.36
	Reading‐memorizing‐reciting (Figure 4e, right)	7.33 ± 0.24	11.33 ± 0.67	4.02 ± 0.61
	Listening‐memorizing‐reciting (Figure 4f, right)	7.63 ± 0.27	12.67 ± 1.12	5.05 ± 0.95
Translation (L1 to L2)	Reading‐memorizing‐translating sentences (Figure 5a, left)	7.81 ± 0.64	13.27 ± 1.53	5.29 ± 0.98
	Listening‐memorizing‐translating sentences (Figure 5b, left)	7.90 ± 0.26	13.46 ± 1.63	5.85 ± 1.21
	Listening‐memorizing‐translating digits (Figure 5c, left)	7.81 ± 0.19	10.35 ± 0.48	2.53 ± 0.43
Translation (L2 to L1)	Reading‐memorizing‐translating sentences (Figure 5a, right)	7.72 ± 0.27	12.23 ± 0.87	4.52 ± 0.87
	Listening‐memorizing‐translating sentences (Figure 5b, right)	8.44 ± 0.39	13.86 ± 1.35	5.44 ± 1.18
	Listening‐memorizing‐translating digits (Figure 5c, right)	7.95 ± 0.22	10.13 ± 0.83	2.10 ± 0.61

### 2.6 Image acquisition

Functional and structural brain images were acquired using Siemens MAGNETOM Prisma 3 T MRI scanner with a 32‐channel head coil at the Centre for Cognitive and Brain Sciences, University of Macau. Each fMRI session consisted of 12 functional scans and 1–2 structural scans, which in total lasted for about 1.5 h. Each functional scan was acquired using a blipped‐CAIPIRINHA simultaneous multislice (SMS), single‐shot echo planar imaging sequence with the following parameters: acceleration factor slice = 5; interleaved ascending slices; TR = 1000 ms; TE = 30 ms; flip angle = 60°; 55 axial slices; field of view = 192 × 192 mm; matrix size = 64 × 64; voxel size = 3 × 3 × 3 mm; bandwidth = 2368 Hz/Px; 256 TR per image; dummy = 6 TR; effective scan time = 256 s. After the sixth functional scan, a set of T1‐weighted structural images (alignment scan) was acquired using an MPRAGE sequence with the same center of slice groups and orientation as the functional images and with the following parameters: TR = 2300 ms; TE = 2.26 ms; TI = 900 ms; Flip angle = 8°; 256 axial slices; field of view = 256 × 256 mm; matrix size = 256 × 256; voxel size = 1 × 1 × 1 mm; bandwidth = 200 Hz/Px; scan time = 234 s. For the first session of each subject, a second set of structural images was acquired with parameters identical to the first set after the last functional scan.

### 2.7 Data preprocessing

For each fMRI session, raw functional images were converted to the Analysis of Functional NeuroImages (AFNI) BRIK format. All BRIK files were registered with the first volume (target) of the seventh BRIK and corrected for motion using AFNI's *3dvolreg* tool. The volume registration process yielded the time series of six degrees of freedom (three translational and three rotational motion parameters; Figure S3). With head restraining measures, including custom‐molded masks and deformable filling inside the head coil, no subject has shown major motion artifacts in functional images (Figure S4).

Bilateral cortical surfaces of each subject were reconstructed from the average of two sets of structural images using FreeSurfer 7.2 (Dale et al., 1999; https://surfer.nmr.mgh.harvard.edu/). All motion‐corrected functional images were registered with the alignment scan (structural images) in each fMRI session, and in turn registered with each subject's cortical surfaces using FreeSurfer‐compatible *csurf* (Sereno et al., 2022; https://pages.ucsd.edu/~msereno/csurf/; https://mri.sdsu.edu/sereno/csurf/), which includes tools for functional image analyses as detailed below.

### 2.8 Functional image analyses

**FIGURE 3:**
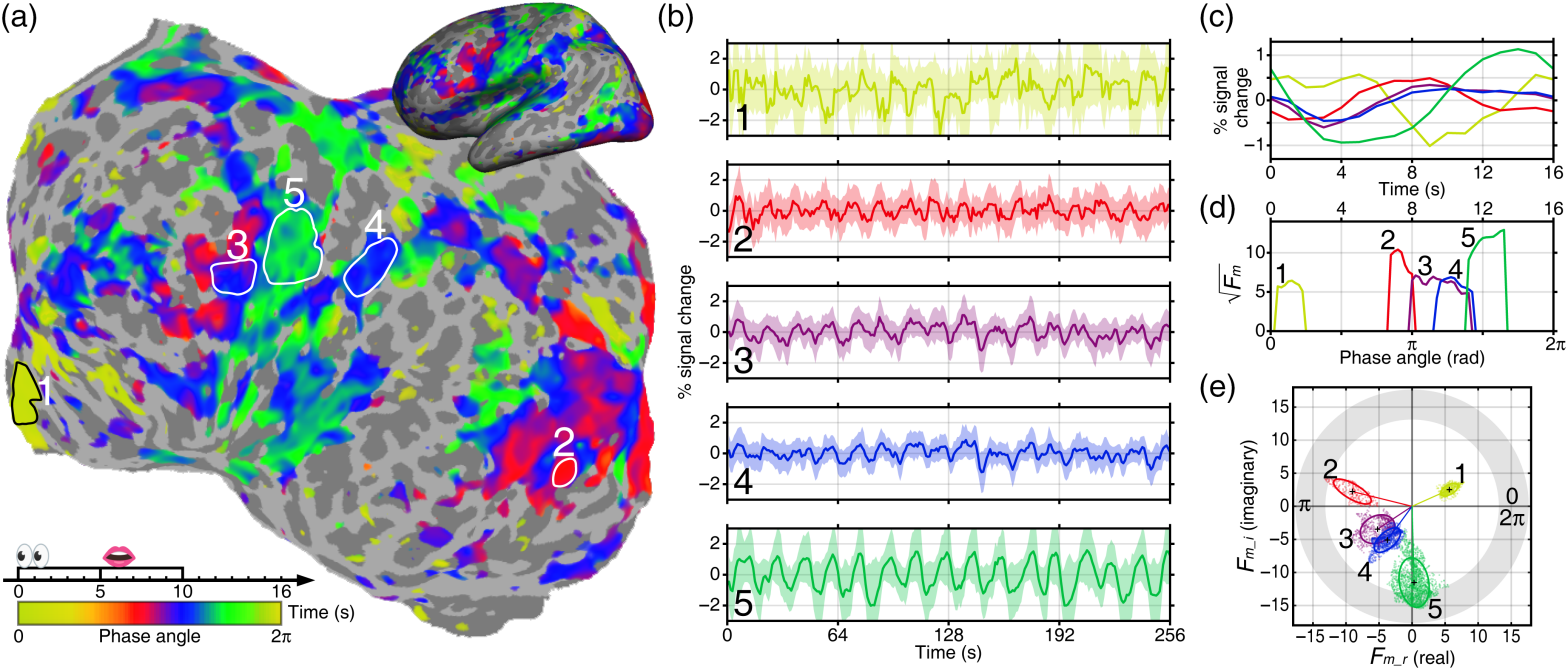
Comparing phase distributions between brain regions. (a) A single‐subject phase‐encoded activation map of a Chinese reading‐memorizing‐reciting task. Five surface‐based regions of interest (sROIs) were selected based on sorted phases (1: Medial prefrontal cortex; 2: visual areas V4v and V8; 3: ventral premotor cortex; 4: inferior parietal lobe; 5: primary sensorimotor cortex). (b) Raw and average blood oxygen level dependent (BOLD) time courses over the scan duration (256 s) for voxels enclosed in each sROI. (c) Average BOLD signal change over one cycle (16 s) for each sROI. Each curve corresponds to same‐color time courses in (b). (d) Surge profile of each sROI where the *y*‐axis indicates the magnitude of activations (statistical significance), and the x‐axis indicates the phase within a cycle [0–2π] that corresponds to a time delay between 0 and 16 s. (e) Distribution of activation amplitudes and phases in the complex plane for five sROIs.

For each BRIK file of a functional scan containing (*x*, *y*, *z*, *t*) = 64 × 64 × 55 × 256 data points, a 256‐point discrete Fourier transform was applied to the time series *x*_*m*_(*t*) of each voxel *m* at location (*x*, *y*, *z*) by:

(1) $$X_{m}\left(\omega \right)=\sum_{t=1}^{256}x_{m}\left(t\right)e^{-\mathrm{j\omega t}}=\left|X_{m}\left(\omega \right)\right|e^{j\theta_{m}\left(\omega \right)},$$

where *X*(*ω*) is the Fourier component at each frequency *ω* between 0 and 127 cycles per scan, and |*X*_*m*_(*ω*)| and *θ*_*m*_(*ω*) represents the amplitude and phase angle, respectively. The task frequency is defined as *ω*_s_ (16 cycles per scan), denoting the frequency of periodic fluctuations of blood flow in response to periodic stimuli and tasks. The remaining nontask frequencies are defined as *ω*_n_. The signal and noise are defined as the Fourier components *X*_*m*_(*ω*) at frequencies *ω*_s_ and *ω*_n_, respectively. The statistical significance of periodic fluctuations of blood flow is evaluated by the signal‐to‐noise ratio, an *F*‐ratio (Chen et al., 2019; Huang et al., 2012, 2017; Sereno et al., 1995, 2001; Sereno & Huang, 2006; Sood & Sereno, 2016), in each voxel *m* by:

(2) $$F_{m}=\frac{{\left|X_{m}\left(\omega_{s}\right)\right|}^{2}/df_{s}}{\left(\sum_{\omega_{n}}{\left|X_{m}\left(\omega_{n}\right)\right|}^{2}\right)/df_{n}},$$

where *df*_s_ = 2 and *df*_n_ = 230 are the degrees of freedom of the signal and noise, respectively. The *p*‐value in each voxel *m* is estimated by the cumulative distribution function *F*_(2,230)_ = *F*(*F*_*m*_; *df*_s_, *df*_n_) (Chen et al., 2019; Huang et al., 2012, 2017; Press et al., 1992). A complex *F*‐value, (*F*_*m_r*_, *F*_*m_i*_), incorporating both the *F*‐statistic value and the phase angle, *θ*_*m*_(*ω*_s_), of each voxel was computed by *F*_*m_r*_ = *f*_*m*_ cos(*θ*_*m*_(*ω*_*s*_)) and *F*_*m_i*_ = *f*_*m*_ sin(*θ*_*m*_(*ω*_s_)), where *f*_*m*_ is the square root of *F*_*m*_. Voxels containing strong periodic signals at the task frequency (*ω*_s_ = 16 cycles per scan) with *F*_(2,230)_ > 4.7 (*p* < .01, uncorrected), *F*_(2,230)_ > 7.1 (*p* < .001, uncorrected), or *F*_(2,230)_ > 9.6 (*p* < .0001, uncorrected) were retained and their phase angles were color‐coded in a range between 0 and 2π (0–16 s) and painted on each individual subject's cortical surfaces for each scan using *csurf* (Figure 3).The complex *F*‐values of corresponding voxels *m* were vector‐averaged (voxel‐wise) across two scans *k* = {1, 2} of the same task in each session for each subject *S* using:

(3) $${\bar{F}}_{m\_S}=\frac{1}{2}\sum_{k=1}^{2}\left(F_{m\_r\_k},F_{m\_i\_k}\right),$$

which was performed by the “Combine 3D Phase Stats” function of *csurf*. The resulting average complex *F*‐values $\left({\bar{F}}_{m\_S\_r},{\bar{F}}_{m\_S\_i}\right)$ were then painted on individual subject's cortical surfaces.

**FIGURE 4:**
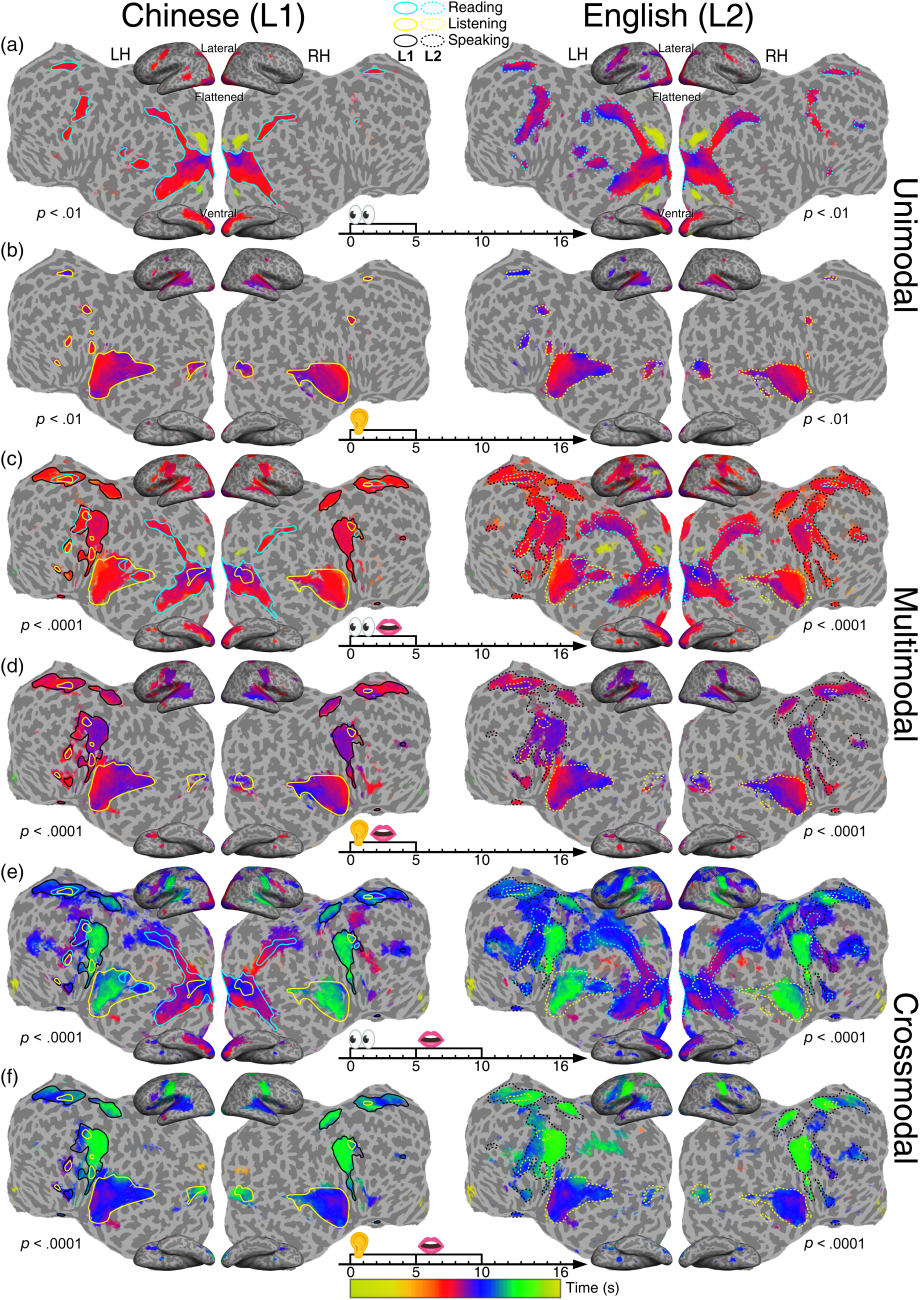
Group‐average maps (*n* = 21) of periodic activations in single‐language tasks. (a) Silent reading (0–5 s). (b) Listening (0–5 s). (c) Reading aloud (0–5 s). (d) Shadowing (listening to and immediately repeating a speech within 0–5 s). (e) Reading and memorizing a written sentence (0–5 s) then reciting it (5–10 s). (f) Listening to and memorizing a spoken sentence (0–5 s) then reciting it (5–10 s). Significant periodic activations are displayed on the inflated (lateral and ventral) and flattened cortical surfaces of a single subject. L1: Chinese; L2: English. LH, left hemisphere; RH, left hemisphere. The colorbar indicates the phase angles (0–2π) of blood oxygen level dependent signals corresponding to a trial period (0–16 s). A lower statistical significance threshold (*F*_[2,230]_ > 4.7; equivalent *p* < .01, uncorrected) is set for the maps of unimodal (reading and listening) tasks to match the spatial extent of significant activations (*F*_[2,230]_ > 9.6; equivalent *p* < .0001, uncorrected) in multimodal and crossmodal tasks. Color contours indicate regions activated by silent reading (cyan), listening only (yellow), and speaking (black). Solid contours: L1 activations; dotted contours: L2 activations.

**FIGURE 5:**
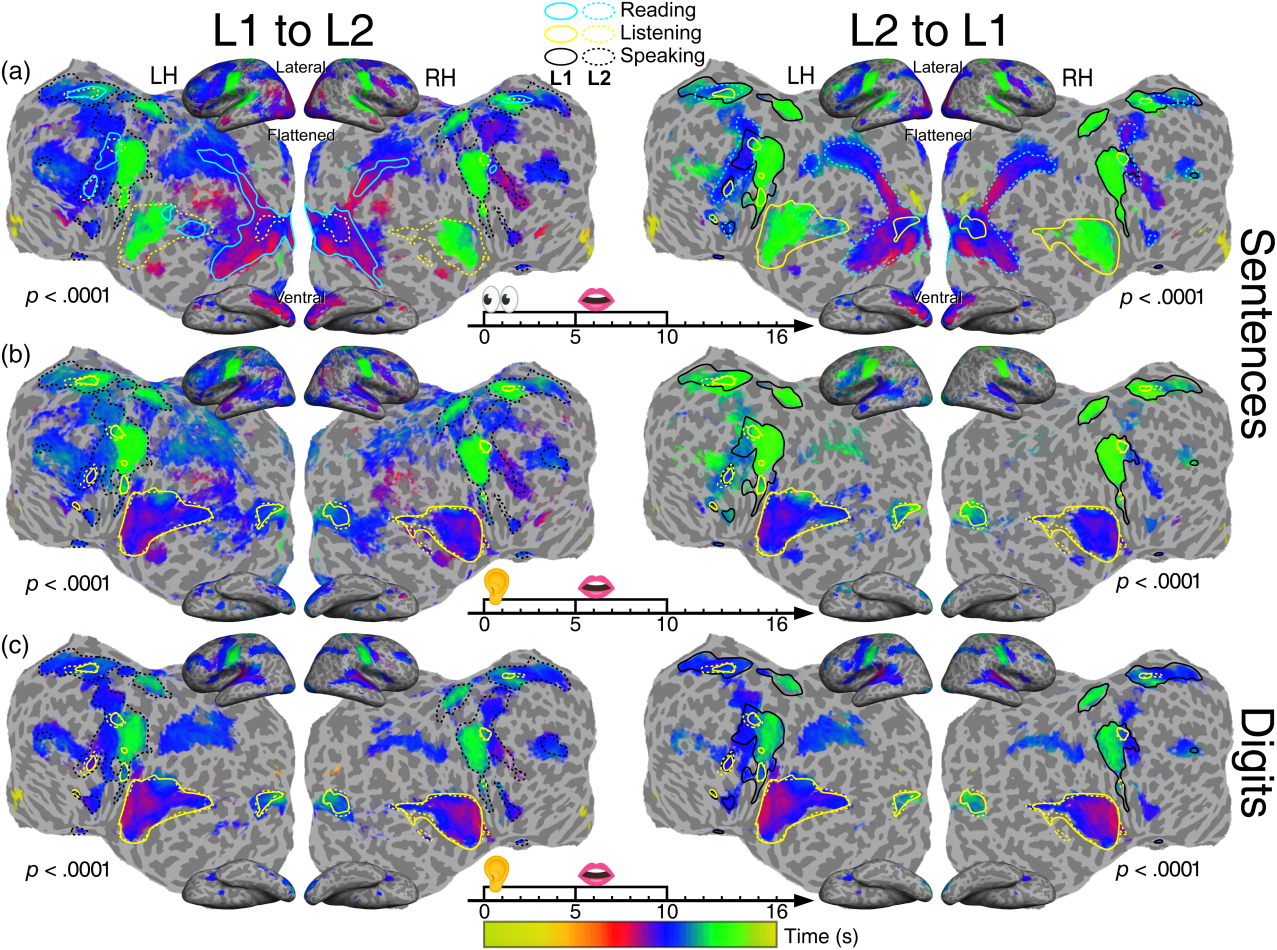
Group‐average maps (*n* = 21) of periodic activations in translation tasks. (a) Text‐to‐speech translation (reading and memorizing a written sentence in 0–5 s, then verbalizing it in the target language in 5–10 s). (b) Speech‐to‐speech translation (listening to and memorizing a sentence in 0–5 s, then vocalizing it in the target language in 5–10 s). (c) Digit interpreting (listening to and memorizing five digits in 0–5 s, then vocalizing them in the target language in 5–10 s). All maps are displayed with a statistical significance *F*_[2,230]_ > 9.6 (equivalent *p* < .0001, uncorrected). LH, left hemisphere; RH, left hemisphere. L1: Chinese; L2: English. Solid and dotted contours represent unimodal L1 and L2 activations outlined in Figure 4.

To find consistent spatiotemporal activation patterns across subjects, surface‐based group averaging methods (Chen et al., 2019; Fischl et al., 1999; Hagler et al., 2006; Huang et al., 2012; Sereno et al., 2022; Sereno & Huang, 2006; Sood & Sereno, 2016) (*csurf* “Cross Sess SphereAvg” function) were used to resample all surface maps containing single‐subject average complex *F*‐values to a common spherical coordinate system for each hemisphere. The complex *F*‐values at each vertex *v* on the common spherical surface were vector‐averaged (vertex‐wise) across all subjects (*N* = 21) using:

(4) $${\bar{F}}_{v\_G}=\frac{1}{N}\sum_{S=1}^{N}\left({\bar{F}}_{m\_S\_r},{\bar{F}}_{m\_S\_i}\right),$$

which yielded a map of group‐average complex values $\left({\bar{F}}_{v\_G\_r},{\bar{F}}_{v\_G\_i}\right)$ for each language task. Finally, the resulting group‐average complex *F*‐values in the common spherical surface were projected back to the cortical surface of any single subject selected, and visualized as phase‐encoded (complex‐number) activation maps via the *csurf tksurfer* interface (Figures 4 and 5).

### 2.9 Static and dynamic brain “weather” maps

**FIGURE 6:**
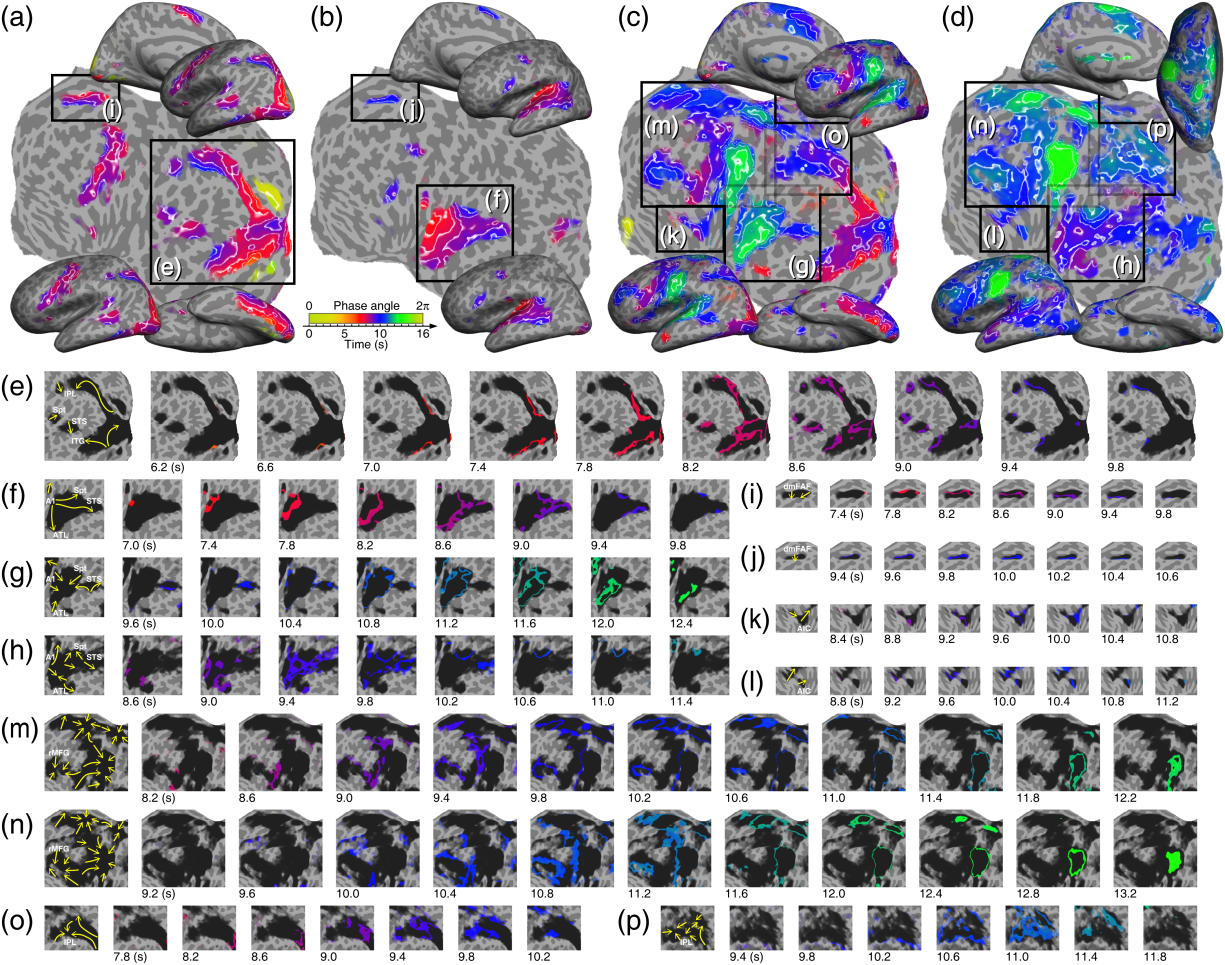
Tracking the paths of traveling waves on isophase contour maps. (a) L2 reading task (Figure 4a, right). (b) L2 listening task (Figure 4b, right). (c) L1 reading‐memorizing‐reciting task (Figure 4e, left). (d) L1 (listening) to L2 (verbalizing) interpreting task (Figure 5b, left). (e–p) Each frame sequence shows the traveling waves (cf. storm rainbands) in a corresponding box in (a–d). The isophase of traveling waves in each frame is color‐coded and displayed over dark “clouds” representing the extent of activations in (a–d). Arrows in the leftmost frame of each sequence represent the paths of local traveling waves. See Figure S5–S22 for high‐resolution vector field maps.

To unfold the complex spatiotemporal patterns in phase‐encoded maps, we created isophase contour maps to illustrate the directions and wavelengths of traveling waves on flattened cortical surfaces (Figure 6). Vertices containing the same range of phase angles (i.e., isophase) were painted with white “surf line” contours by setting the $phasecontourflag = 1 in *csurf tksurfer* (Figure 6a–d). To find the propagation paths of traveling waves, a vector field map of phase gradient (cf. maps of wind or ocean current patterns) was estimated using a nearest neighbor vertex basis on the flattened cortical surface (*tksurfer compute_surfgrad* tool). The resulting principal phase gradient directions are indicated by arrows (paths) in the leftmost panel of each movie frame sequence in Figure 6e–p. Detailed vector field maps are shown in Figures S5–S22 and Movies [Link], [Link].

In addition to static maps of isophase contours and phase gradient, we created a traveling wave (“brainstorm”) movie for each language task using the *tksurfer phasemovie.tcl* script to illustrate the dynamic traveling waves (cf. animated “rainbands” of an impending storm; see Movies [Link], [Link]). Each frame of the movie shows surface vertices containing periodic activations with phase angles falling in a moving window (range = 9°; step = 4.5°, equivalent to 0.2 s/frame) at each moment. The isophase activation patterns were rendered in phase‐encoded colors using the colorbar in Figures 4 and 5. In total, each movie contains 80 frames of spatiotemporal traveling waves within a period of 0–16 s (corresponding to [0, 2π] or [0°, 360°]).

**FIGURE 7:**
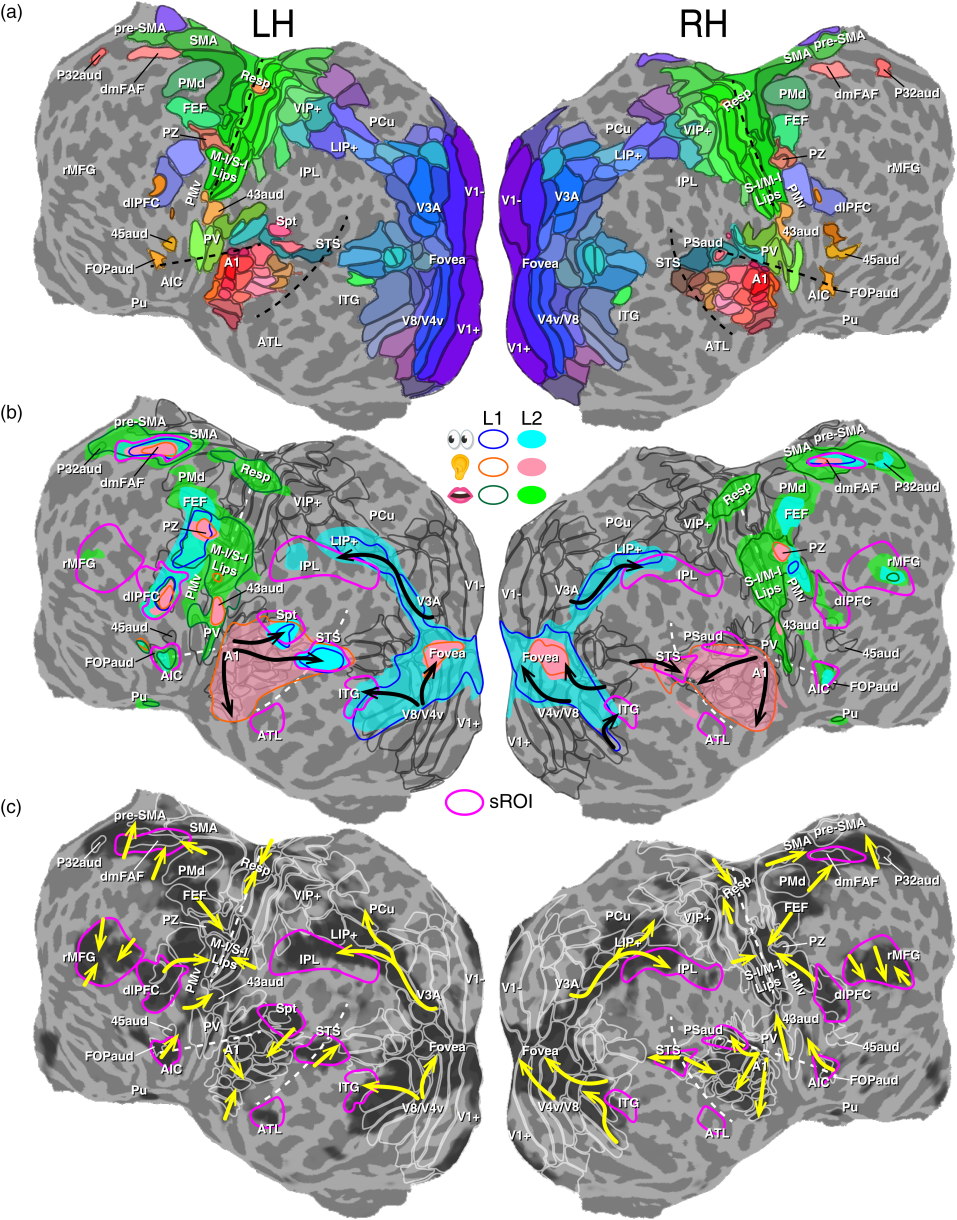
Tracking traveling waves and surges on multilayer brain “weather” maps. (a) Topological maps with dark gray contours indicating the borders of visual, auditory and somatomotor areas displayed on the flattened cortical surfaces of a single subject (Table 2; see Sereno et al., 2022 for a full list of brain areas). (b) Multilayer single‐language activation maps overlaid on topological areas. Color contours (L1) and filled regions (L2) outline the extent of unimodal (reading and listening) or multimodal (speaking and self‐monitoring) activations in Figure 4. Black arrows indicate multiple streams of traveling waves in visual and auditory cortices. (c) Tracking the paths (yellow arrows) of regional traveling waves over topological areas (white contours) during a L1 reading‐memorizing‐reciting task (Figure 4e, left; see Figure S9 for a high‐resolution vector field map). Dark “clouds” represent the extent of significant periodic activations (*F*_[2,230]_ > 7.1; equivalent *p* < .001, uncorrected). Magenta contours indicate surface‐based regions of interest (sROIs) of language and domain‐general cognitive regions outside or partially overlapping topological areas. AIC, anterior insular cortex; ATL, anterior temporal lobe; A1, primary auditory cortex; dlPFC, dorsolateral prefrontal cortex; dmFAF, dorsomedial frontal auditory field; FEF, frontal eye fields; FOPaud, frontal operculum auditory; IPL, inferior parietal lobe; ITG, inferior temporal gyrus; LIP+, lateral intraparietal areas; M‐I, primary motor cortex; PCu, precuneus; PMd, dorsal premotor cortex; PMv, ventral premotor cortex; pre‐SMA, pre‐supplementary motor area; Pu, putamen; PV, parietal ventral somatosensory area; PZ, polysensory zone; P32aud, area p32, auditory part; Resp, respiratory area; SMA, supplementary motor area; STS, superior temporal sulcus; Spt, Sylvian parietal temporal area; S‐I, primary somatosensory cortex; rMFG, rostral middle frontal gyrus; VIP+, ventral intraparietal areas; V1+/V1−, V3A, V8, V4v, visual areas; 43aud, subcentral auditory area; 45aud, auditory area 45.

Multilayer brain maps were constructed to facilitate interpretation of the complex spatiotemporal patterns of traveling waves and comparison across different language tasks (Figure 7). The bottom layer of Figure 7 contains flattened cortical surfaces of the left and right hemispheres reconstructed from the structural brain images of a single subject. This layer shows the anatomy (cf. terrain) of the cortical surface, with dark‐gray regions representing sulci (cf. valleys) and light‐gray regions representing gyri (cf. ridges). To navigate in “uncharted territory” on the cortical surface, a recent brain atlas outlining the latest parcellation of topological visual, auditory, and somatomotor maps (Sereno et al., 2022; https://pages.ucsd.edu/~msereno/csurf/fsaverage-labels/) was mapped onto the cortical surfaces of this subject using the *csurf tksurfer annot2roi.tcl* script (Figure 7a). The contours (gray) of topological areas were overlaid on flattened cortical surfaces in a way similar to the “county” lines in a weather forecast map (Figure 7b). This second layer was then used as a reference map (e.g., “landmarks” of regions of interest [ROIs]) to guide the interpretation of functional activations on the maps of unimodal, multimodal, crossmodal, and cross‐language tasks. To this end, we overlaid six layers of language maps containing contours taken from Figure 4, which show the extent of brain activations (cf. clouds in a satellite image) in L1 or L2 reading‐only (unimodal), listening‐only (unimodal), and speaking (multimodal) tasks (Figure 7b). We then illustrated the paths of surface traveling waves (cf. storm paths; Li et al., 2018) during unimodal reading or listening tasks (black arrows in Figure 7b) and during a crossmodal L1 reading‐memorizing‐reciting task (yellow arrows in Figure 7c). Due to different stimulus properties and task requirements, the activation extent in multimodal or crossmodal tasks does not always encompass the full activation extent in unimodal tasks.

### 2.10 Surface‐based ROI analysis

**FIGURE 8:**
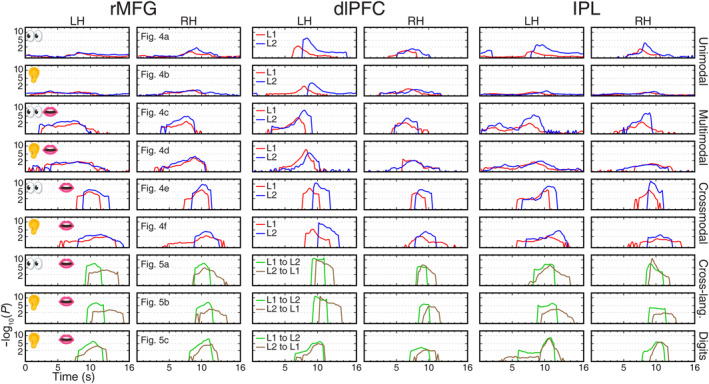
Surge profiles of traveling waves in six bilateral surface‐based regions of interest (sROIs; rostral middle frontal gyrus [rMFG], dorsal lateral prefrontal cortex [dlPFC], and inferior parietal lobe [IPL]). Each row corresponds to a task in Figures 4 and 5 as labeled. The height of surge is indicated by −log_10_(uncorrected *p*‐value) in the *y*‐axis. Red and blue profiles indicate L1 and L2 single‐language tasks, respectively. Green and brown profiles indicate L1‐to‐L2 and L2‐to‐L1 translation tasks, respectively. L1: Chinese; L2: English. LH, left hemisphere; RH, left hemisphere.

**FIGURE 9:**
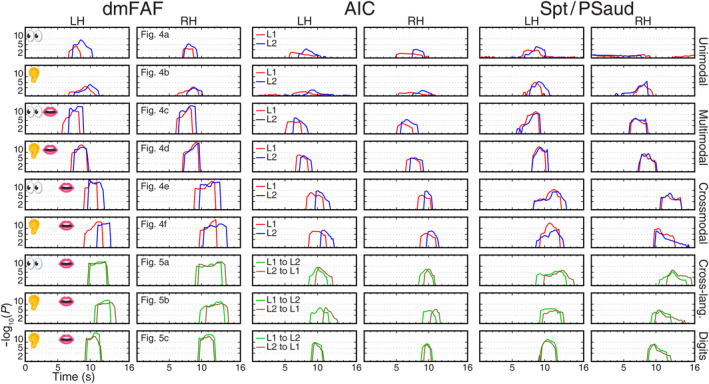
Surge profiles of traveling waves in six bilateral surface‐based regions of interest (dorsomedial frontal auditory field [dmFAF], anterior insula cortex [AIC], and Sylvian parietal temporal area [Spt/PSaud]). All conventions are the same as those in Figure 8.

**FIGURE 10:**
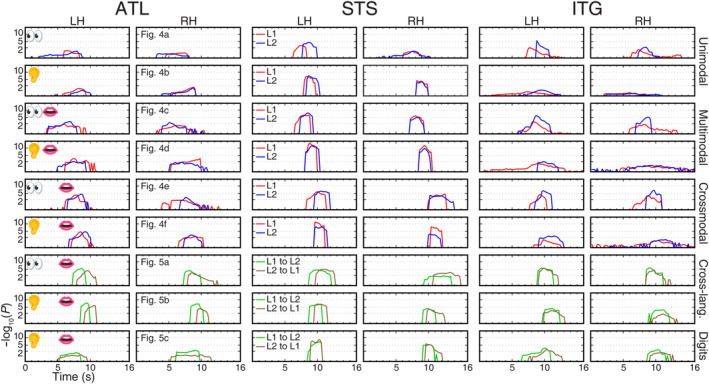
Surge profiles of traveling waves in six bilateral surface‐based regions of interest (anterior temporal lobe [ATL], superior temporal sulcus [STS], and inferior temporal gyrus [ITG]). All conventions are the same as those in Figure 8.

As hemodynamic traveling waves “escaped” from the topological maps, they made “landfall” in language‐specific and domain‐general cognitive regions, which caused transient surges of blood flow in these regions. Through a task‐driven functional‐ROI approach, we selected five language‐related and four domain‐general cognitive surface‐based ROIs (sROIs; magenta contours in Figure 7b,c). Language‐related sROIs were defined based on language mapping literature, regardless of language task or modality (Hickok et al., 2003, 2009; Labache et al., 2019; Price, 2012). Domain‐general cognitive sROIs were areas that played a role in the language network but were not specific to language functioning (Calabria et al., 2018; Hagoort, 2017; Hervais‐Adelman & Babcock, 2020). The borders of these sROIs were outlined with a moderate statistical threshold (*p* < .01, uncorrected) to encompass the larger common regions of activations across different tasks in the group‐average maps. Language‐related sROIs include (i) anterior temporal lobe (ATL), (ii) anterior insula cortex (AIC), (iii) inferior temporal gyrus (ITG), (iv) Sylvian parietal temporal area (Spt), and (v) superior temporal sulcus (STS). Domain‐general cognitive sROIs include (i) dorsomedial frontal auditory field (dmFAF; part of presupplementary motor [pre‐SMA] and supplementary motor areas [SMA], labeled separately as “dorsomedial frontal auditory field” according to Sereno et al., 2022), (ii) dorsal lateral prefrontal cortex (dlPFC), (iii) inferior parietal lobe (IPL), and (iv) rostral middle frontal gyrus (rMFG). These nine sROIs were then used to compare the hemodynamic surge profiles (defined below) of unimodal, multimodal, crossmodal, and cross‐language tasks on the group‐average maps (Figures 8, 9, 10).

For each task, a hemodynamic surge profile (by analogy with storm surge; Li et al., 2018) was estimated from the distribution of group‐average complex *F*‐values (*F*_*v_G_r*_, *F*_*v_G_i*_) of all vertices within each sROI using circular statistics (Chen et al., 2019; Huang et al., 2017). First, the phase angle of each vertex *v* was obtained by *θ*_*v_G*_ = atan2$\left({\bar{F}}_{v\_G\_r},{\bar{F}}_{v\_G\_i}\right)$ in Matlab. Second, the 16‐s period was divided into 80 equally spaced bins between [0°, 360°] and [0, 2π], yielding a temporal resolution of 0.2 s per bin. A total of *V* vertices were found with phase angles *θ*_*v_G*_ falling within the moving window centered at each bin *d* (window size = 9°; step = 4.5°, equivalent to 0.2 s/bin). The complex *F*‐values of *V* vertices in the moving window *d* were averaged using:

(5) $${\bar{F}}_{v\_d}=\frac{1}{V}\sum_{v=1}^{V}\left({\bar{F}}_{v\_G\_r},{\bar{F}}_{v\_G\_i}\right)$$

The magnitude of the average complex *F*‐value was then obtained by:

(6) $$\left|{\bar{F}}_{v\_d}\right|=\sqrt{{\bar{F}}_{v\_d\_r}^{2}+{\bar{F}}_{v\_d\_i}^{2}}$$

A *p*‐value was estimated for each $\left|{\bar{F}}_{v\_d}\right|$ using the cumulative distribution function *F*_(2,230)_. Finally, the height of surge is computed by −log_10_(*p*‐value) as shown in the *y*‐axis of each subplot in Figures 8, 9, 10. The resulting surge profiles reveal the arrival time, ending, latency, and peak magnitude of hemodynamic traveling waves in each sROI (Figures 7c and 8, 9, 10).

### 2.11 University of Macau Brain Atlas

In this study, functional activation maps of unimodal, multimodal, crossmodal, and cross‐language tasks were displayed on the same cortical surfaces, which enable direct comparison of spatiotemporal traveling wave patterns across languages and tasks on multilayer surface‐based functional brain atlases (Huang & Sereno, 2018; Sereno et al., 2022; Sood & Sereno, 2016). To share these results with other researchers, we constructed an open access website, the University of Macau Brain Atlas (UMBA; https://cstic.um.edu.mo/umba), incorporating all maps and movies in this study (Movie S19). This online atlas will undergo continuous updates with new layers of maps and movies of multiple populations, languages, and tasks from our ongoing studies involving more than 150 subjects.

The design of multilayer user interface in UMBA drew on concepts from Google Earth (Movie S20; https://earth.google.com/web/) and weather forecast websites (e.g., time‐lapse view of moving rainbands on https://www.wunderground.com/wundermap/). The UMBA website was developed using HTML, CSS, and Javascript languages. The Vue.js frontend JavaScript framework (version 2.x; libraries: vuetify, vue‐i18n) was used for “responsive reactive design” for the user interface. Built on a pair of flattened cortical surfaces, each of the following layers can be made visible or hidden according to the user's preference: (1) topological maps (Sereno et al., 2022); (2) contours of topological areas; (3) annotation of major sulci; (4) three layers of maps of reading, listening, and speaking tasks for each language (six layers in total); (5) contours of sROI; (6) paths of traveling wave; (7) labels (landmarks) of brain areas and sROI; (8) phase‐encoded activation maps for each task and language; (9) traveling wave (isophase) movie for each task and language. A demonstration of UMBA interface and multilayer maps is shown in Movie S19.

## 3 RESULTS

### 3.1 Static and dynamic brain “weather” maps

Single‐subject and group‐average maps of statistically significant periodic activations at 16 cycles per scan and their phases are displayed in an orange‐red‐blue‐green‐yellow phase gradient on the flattened cortical surfaces of an individual subject (Figures 3, 4, 5). The systematic phase difference between different brain regions is illustrated in Figure 3. The spatiotemporal patterns of surface traveling waves in four representative tasks are illustrated as isophase contour maps (Figure 6). To interpret the traveling wave patterns, we used the topological maps recently published by Sereno et al. (2022) as a reference map for locating and labeling functional brain areas (Figure 7a). For easy comparison of activated areas between different tasks and languages, we overlaid the contours of L1 and L2 modality‐specific activations on the topological maps and summarized the directionality of traveling waves in Figure 6b,c. The visualization of spatiotemporal unfolding of hemodynamic traveling wave patterns in static brain maps and movies (Figures 6 and 7, and S5–S22 and Movies [Link], [Link]) is analogous to the tracking of dynamic rainbands across state and county lines on a dynamic weather radar map. The color‐coded maps here represent surface traveling waves (Sereno et al., 1995, 2001, 2022; Sereno & Huang, 2006; Sood & Sereno, 2016) propagating within and across fixed cortical area borders (cf. county lines) of topological and non‐topological areas during real‐time language processing. Arrows indicate the local direction of the coherent traveling waves in static pictures (Figures 6e–p and 7b,c). Abbreviations of cortical areas are summarized in Table 2.

**TABLE 2:** Abbreviations of cortical areas in alphabetical order.

Abbreviation	Cortical area
AIC	Anterior insular cortex
ATL	Anterior temporal lobe
A1	Primary auditory cortex
dlPFC	Dorsolateral prefrontal cortex
dmFAF	Dorsomedial frontal auditory field
FEF	Frontal eye fields
FOPaud	Frontal operculum auditory
IPL	Inferior parietal lobe
ITG	Inferior temporal gyrus
LIP+	Lateral intraparietal areas
M‐I	Primary motor cortex
PCu	Precuneus
PMd	Dorsal premotor cortex
PMv	Ventral premotor cortex
pre‐SMA	Pre‐supplementary motor area
Pu	Putamen
PV	Parietal ventral somatosensory area
PZ	Polysensory zone
P32aud	Area p32, auditory part
SMA	Supplementary motor area
STS	Superior temporal sulcus
Spt	Sylvian parietal temporal area
S‐I	Primary somatosensory cortex
rMFG	Rostral middle frontal gyrus
VIP+	Ventral intraparietal areas
V1+/V1−, V3A, V8, V4v	Visual areas
43aud	Area 43, subcentral area, auditory
45aud	Area 45, auditory

### 3.2 Reading phase‐encoded activation maps

Figure 3 demonstrates the systematic phase differences between brain regions that were involved in different phases (color‐coded) of sequential language processing during a Chinese (L1) reading‐memorizing‐reciting task (Figure 2e) by a single subject. The slow hemodynamic response resulted in ~6 s delays of brain activations in regions involved in reading (reddish), encoding and memory (bluish), motor planning (purplish), speech output and self‐monitoring (greenish), and resting state (yellowish; Figure 3a). Owing to the periodic nature of the phase‐encoded design where the subject was required to read and memorize written sentences, recite, and rest periodically for 16 times per scan, the averaged BOLD time courses reflected the periodic activations by having 16 pairs of peak and trough (Figure 3b). For each region, the average time courses in a 16‐s cycle were further averaged across 16 trials per scan (Figure 3c), which clearly showed different activation timing between regions. The distribution of activation phases in each region is shown in Figure 3d,e. Because of parallel activations, the distribution of phase angles between different brain regions partially overlapped.

### 3.3 Visualizing surface traveling waves

Figure 4 shows the phase‐encoded activation maps of unimodal (involving a single sensory modality), multimodal (involving two or more sensory and motor modalities concurrently), and crossmodal (changing modalities) tasks in Chinese or English. Multimodal and crossmodal tasks always elicited more extensive activations than unimodal tasks. Figure 5 shows the phase‐encoded activation maps for six translation tasks. For sentence translation, the L1‐to‐L2 direction (Figure 5a,b) always induced slightly larger activations than the L2‐to‐L1 direction. Similarly, for digit translation, the L1‐to‐L2 direction (Figure 5c) induced a slight increase in activated areas compared with the L2‐to‐L1 direction.

To assist interpretation of the intrinsic spatiotemporal brain dynamics during unimodal, crossmodal, and cross‐language tasks, we produced a series of isophase movies illustrating the paths of traveling waves across flattened cortical surfaces (Movies [Link], [Link]). For example, in the L1 reading‐memorizing‐reciting task (Figures 4e and 6c and Movie S5), waves of activations first emerged in dorsal and ventral visual areas and ATL during reading. The occipital waves then branched off into dorsal and ventral visual streams, reaching up to lateral intraparietal areas (LIP+) and ITG, respectively. Around the time when the waves reached LIP+ and ITG, the premotor cortex, AIC, dlPFC, and rMFG were activated. As the waves from the premotor cortex and AIC continued to travel toward oral sensorimotor areas, the pre‐SMA was also activated. The waves then propagated from pre‐SMA to SMA in preparation for speech output. Finally, as the subjects started vocalizing, the sensorimotor and auditory cortices were activated simultaneously, reflecting self‐monitoring during speech production.

The activation patterns in each movie corresponding to a language task (Figures 4 and 5) are described in detail below. In reading tasks (Figure 4a and Movies S1 and S7), activation waves emerged from early visual areas, and branched off into the dorsal and ventral visual streams (Huang & Sereno, 2013, 2018; Sereno et al., 2022; Sood & Sereno, 2016; Ungerleider & Haxby, 1994), reaching up to LIP+ and ITG (Figures 6a,e and 7b). Smaller clusters of activations were also present in the STS, Spt (Hickok et al., 2003, 2009), an area overlapping both pre‐SMA and SMA (labeled separately as dmFAF; Sereno et al., 2022), dlPFC, frontal eye fields, and premotor cortex. In listening tasks (Figure 4b and Movies S2 and S8), activation waves emerged from the primary auditory cortex (A1), and branched off into three streams (Hickok & Poeppel, 2007; Rauschecker & Scott, 2009), reaching: (1) superior temporal gyrus (STG) and Spt, (2) STS, and (3) ATL (Figures 6b,f and 7b). Other smaller clusters of activations were observed in pars triangularis, dlPFC, auditory polysensory zone, area 43aud, and SMA (Figure 7b). On the whole, the activations were predominantly bilateral for both English and Chinese reading and listening tasks.

Multimodal reading‐aloud tasks (Figure 4c and Movies S3 and S9) involved reading, speaking, and self‐monitoring of speech production simultaneously. Correspondingly, additional activations as compared with reading (Figure 4a and Movies S1 and S7) and listening tasks (Figure 4b and Movies S2 and S8) were found in SMA, primary and secondary sensorimotor cortex (oral and diaphragm representations), pars triangularis, STG, and AIC (outlined by black contours in Figure 4c). Multimodal shadowing tasks (Figure 4d and Movies S4 and S10) involved listening to stimuli, speaking, and self‐monitoring simultaneously. Additional activations were found in pre‐SMA, sensorimotor cortex, and AIC as compared with listening tasks (Figure 4b). Compared with reading‐aloud tasks (Figure 4c), shadowing tasks activated broader areas in the STS bilaterally. Although the subjects were instructed to speak as soon as the stimuli appeared, delays in activation (purplish) were found around the premotor and sensorimotor areas in shadowing tasks (Figure 4d) for both English and Chinese. The audio recordings confirmed that shadowing tasks induced delays in speech onset and offset compared with reading‐aloud tasks (Table 1). Similar to the unimodal maps, activations were predominately bilateral (see laterality index in Table S3; Seghier, 2008) and the L2 maps show slightly increased activated areas compared with the L1 maps (Figure 4d).

Crossmodal maps (Figure 4e,f) show sequential activations before, during, and after modality change, indicating that the subjects had to first read silently or listen to the English or Chinese sentences, commit them into memory, and then recite them. In reading‐memorizing‐reciting tasks (Figures 4e and 6c and Movies S5 and S11), the three modalities (reading, speaking, and listening to own speech) each sequentially activated specific brain regions as revealed by the traveling waves, with the early visual areas being first activated (reddish regions). Purple‐bluish regions indicate intermediate activations, which were mainly involved during the modality switch (between reading and speaking). Additional activated areas as compared with reading‐aloud tasks (Figure 4c) include ATL, rMFG, and IPL (Figures 6c,m,o and 7c). Finally, greenish regions indicate late activations (Figure 6c,g,m), which were mainly clustered in the primary sensorimotor areas (oral and diaphragm), auditory cortex, and Spt as the tasks involved speech motor control and self‐monitoring. The smaller extent of activation in the auditory cortex suggests its involvement in hearing and monitoring of self‐speech (Figure 4e; yellow outline). Similar to the unimodal and multimodal maps, the crossmodal activations were bilateral (Table S3). Compared with L1 maps, L2 maps overall show significantly larger extent in activation for the reading‐memorizing‐reciting tasks (Figure 4e; right panel). On the other hand, during the listening‐memorizing‐reciting tasks (Figure 4f and Movies S6 and S12), A1 and ATL were the first areas to be activated. Intermediate activations included higher‐level auditory cortex, AIC, premotor, and pre‐SMA regions. Late activations included fovea, IPL, Spt, and the primary sensorimotor areas. The L1 and L2 maps demonstrate similar patterns except for the activations in the premotor and IPL regions, where activations induced by L2 tasks are larger than those associated with L1 tasks.

Figure 5 shows the spatiotemporal patterns of cross‐language control in translation tasks. The maps were organized by stimulus type (sentences or digit sequences) and translation direction (L1‐to‐L2 or L2‐to‐L1). Color contours were taken from the activations found in reading only, listening only, and speaking (reading aloud) tasks (Figure 4a–c). All activation patterns in the reading‐memorizing‐reciting tasks were found in the reading‐memorizing‐translating tasks (Figure 5a and Movies S13 and S16). Similarly, activations in the listening‐memorizing‐reciting tasks were also present in the listening‐memorizing‐translating tasks (Figure 5b and Movies S14 and S17).

In sentence‐level translation tasks, the L1‐to‐L2 direction involved extensive activations in the posterior middle temporal gyrus (MTG), posterior STS, IPL, dlPFC, rMFG, and superior frontal gyrus. On the other hand, L2‐to‐L1 translation tasks exhibited smaller activation extent in domain‐general cognitive regions and very sparse activations in the posterior MTG and STS. In addition, when compared with the single‐language crossmodal tasks, the cross‐language processing exhibited more extensive activations in ATL, posterior MTG/STS, and the domain‐general cognitive regions, including IPL and prefrontal regions. In digit‐level translation tasks, the L1‐to‐L2 direction slightly expanded the surface area of the activated regions compared with L2‐to‐L1 digit translation (Figure 5c and Movies S15 and S18). In addition, the extent of activations in the STS was similar to those during reading‐aloud and reading‐memorizing‐reciting tasks (Figure 4c,e). Besides a smaller activation in STS, ATL and posterior MTG/STS were not activated in digit‐level translation tasks (Figure 5c) as compared with the verbal sentence‐level translation tasks (Figure 5b), which may help confirm the roles of STS, ATL, and posterior MTG/STS in semantic processing and verbal reasoning. Finally, activations were found in IPL, rMFG, and dlPFC, suggesting the involvement of working memory in translation.

### 3.4 Analyzing local traveling wave patterns

One goal of this study is to present a new way to analyze and visualize spatiotemporal interactions within and across the sensory, motor, language, and cognitive brain regions. We selected four representative tasks to illustrate the complex spatiotemporal patterns of local traveling waves (Figure 6a–d as Movies [Link], [Link], [Link], and S14, respectively). Figure 6a,e shows three streams of waves in the ventral and dorsal visual pathways during an L2 reading task. Two waves emerged from areas V8 and V4v, with one propagating to ITG and the other to foveal representations in early visual areas. The third wave emerged from area V3A and traveled upstream along the LIP+ cluster to reach IPL. All tasks that involved reading triggered and displayed similar patterns and directions of traveling waves in ventral and dorsal visual pathways (Figures 4a,c,e and 5a). Figure 6b,f shows that activation waves emerged from A1 and split into at least three streams reaching ATL, Spt, and STS in the left hemisphere during an L2 listening task. Interestingly, during the production of speech from visually presented sentences such as the reading‐memorizing‐reciting tasks (Figures 4e) and reading‐memorizing‐translating tasks (Figure 5a), the direction of waves emerging from A1 remained the same, while the other two streams showed reverse directions, that is, they initiated from Spt and STS and propagated anteriorly (compare Figure 6f,g).

Regarding domain‐general cognitive regions, traveling waves initiated from the perimeter of rMFG and propagated radially inwards (Figure 6m,n). Similar radial‐inward flow patterns were found in IPL (Figure 6o,p). These patterns remained identical across all the tasks that recruited rMFG and IPL. Similarly, the patterns in AIC and dlPFC remained identical for all the tasks that recruited these regions (Figure 6k,l). Most remarkably, an alignment of planar wavefronts emerged from AIC and dlPFC, which then merged at the border between premotor and sensorimotor areas (Figure 6k–n). Furthermore, distinct spatiotemporal wave patterns were observed in dmFAF: one downward stream was found in the unimodal tasks (Figure 6i,j); two radial‐inward streams that each emerged from the perimeter and propagated towards the center were observed in the multimodal and crossmodal tasks (Figure 6c,d,m,n).

### 3.5 Regional surges of traveling waves

Once the paths of traveling waves were identified, we then selected nine sROIs in domain‐general cognitive regions and language‐related regions, including rMFG, dlPFC, IPL, dmFAF, AIC, Spt, ATL, STS, and ITG (magenta contours in Figure 7b,c), to compare their involvement in the unimodal, multimodal, crossmodal, and cross‐language tasks. Figures 8, 9, 10 show the distribution of phase‐sorted complex *F*‐values within each sROI, which reveals the surge, subside, latency, and peak magnitude of the traveling waves (cf. regional storm surges) of corresponding tasks in Figures 4 and 5. For example, dlPFC was predominantly activated in the left hemisphere across all tasks. The highest activation surges occurred during the crossmodal and sentence translation tasks. Three different latencies were found across all tasks. For the unimodal tasks and digit translation tasks, there were medium activation delays. The delays were short for both of the multimodal tasks. The crossmodal and sentence translation tasks showed the longest delay. L1 production exhibited shorter latency than L2 production while L1‐to‐L2 translation exhibited slightly shorter latency than L2‐to‐L1 translation.

### 3.6 A logistics model of language processing

**FIGURE 11:**
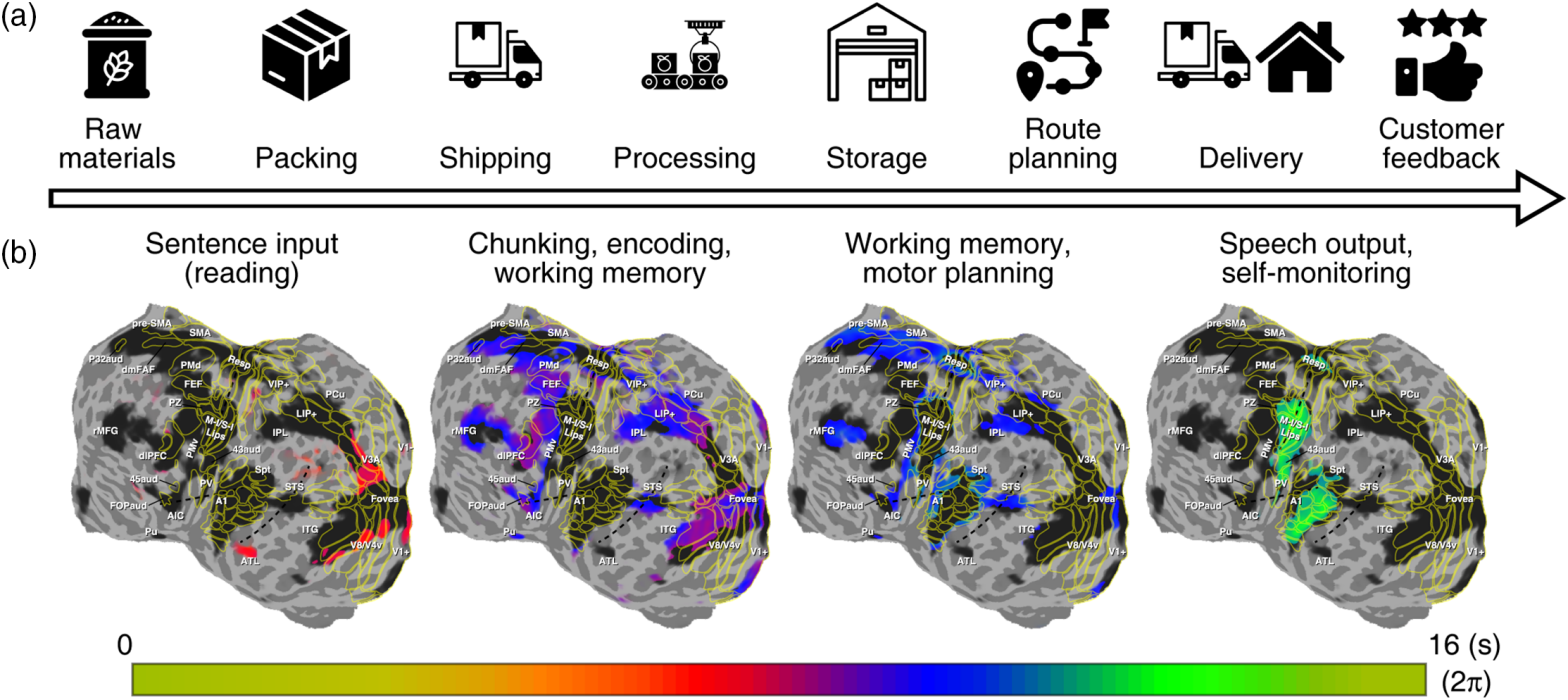
Logistics models. (a) A flowchart for logistics activities. Icons have free licenses and were downloaded from Flaticon (https://www.flaticon.com/). (b) A logistics model for language processing where sentence input mirrors the raw materials stage in (a); chunking a string of information for encoding and memory mirrors the packing, shipping, and processing stages in (a); converting information in working memory to speech and motor planning mirror the storage and route planning stages in (a); and speech output and self‐monitoring mirror the delivery and customer feedback stages in logistics activities.

The timing and location of traveling waves enable us to build a step‐by‐step logistics model of language processing that mirrors a typical logistics flowchart (Figure 11a; Zarbakhshnia et al., 2020). Using the crossmodal reading‐memorizing‐reciting task as an example, we divided the entire process into approximately four stages based on activation phases (Figure 11b). During the stage of sentence input (reddish regions), written information activated the visual systems (V3A, V3v, V4v, and V8) and ATL. As a series of characters/words were chunked into “packages” and carried along the higher‐level visual streams (purplish regions), brain areas involved in working memory and other domain‐general cognitive functions were also activated (dlPFC, outer edges of IPL and rMFG). Once all the information was encoded and memorized, the brain converted the stored information into motor sequence for speech production. The stage of working memory and motor planning (bluish regions) involved brain areas rMFG, AIC, pre‐SMA, SMA, IPL, STS, Spt, and the premotor cortex (PMd and PMv). Finally, during the stage of speech output and self‐monitoring (greenish regions), the sensorimotor cortex (M‐I and S‐I) and the auditory cortex were activated.

## 4 DISCUSSION

Temporal and spatial measures must be combined to acquire a deeper understanding of the neural basis of language processing. Previous time‐resolved fMRI studies on language processing have revealed sequential activations of multiple isolated brain regions, but without showing continuous spatial and temporal progression across regions (Humphries et al., 2007; Janssen & Mendieta, 2020; Reverberi et al., 2015; Sigman et al., 2007). While other existing fMRI studies have demonstrated the spatiotemporal progression of neural activities at resting state, the propagation of task‐evoked (event‐related) activities corresponding to distinct stimulus and response phases were not shown (Bolt et al., 2022; Gu et al., 2021; Pines et al., 2023; Raut et al., 2021). How activation arises in one brain region and propagates toward the next region during real‐time language processing has remained obscure. In visual neuroscience, phase‐encoded fMRI mapping using time‐delayed retinotopic stimuli reveals spatially and temporally continuous traveling waves across the cortical surface (Engel, 2012; Engel et al., 1994; Sereno et al., 1995, 2001, 2022; Sereno & Huang, 2006; Sood & Sereno, 2016). However, the rate of visual, auditory, or tactile stimulation (e.g., a period of 64 s) in typical phase‐encoded designs is too slow for mapping real‐time language perception and production, where a sentence is usually processed within a few seconds. In this study, we developed rapid phase‐encoded fMRI to capture and visualize fine‐grained spatiotemporal BOLD signals propagating over the cortical surface during real‐time naturalistic unimodal, crossmodal, and cross‐language processing. We demonstrated that 5 s of naturalistic reading or listening induced coherent surface waves that traveled continuously across the visual or auditory streams, which had not been revealed in previous time‐resolved fMRI studies of higher cognitive processes (Formisano et al., 2002; Humphries et al., 2007; Janssen & Mendieta, 2020; Reverberi et al., 2015; Sigman et al., 2007). The tracking of traveling waves in isophase movies, showing the brain activity in action, opens a new window into the understanding of the dynamic functional and effective connectivity across the whole brain during real‐time language processing. The timing, location, and direction of traveling waves reveal information flows within and across visual, auditory, sensorimotor, language, and cognitive regions, which can be integrated into existing models of human information processing. Major findings of our study are discussed below.

Across all the experimental tasks for this study (Figures 4a–f and 5a–c), we identified a bilateral network of task‐induced activations during natural language processing, with certain tasks such as consecutive interpretation of sentences exhibited a slight bias to the left hemisphere. It is a long‐standing view that language is predominantly left lateralized, though it has been suggested that the right hemisphere also has a role to play in language processing. Findings from functional neuroimaging studies regarding language lateralization are contradictory. This study gives the most direct and consistent support to the recently proposed bilateral model of language processing, which suggests that the language network is asymmetric (Table S3) but intrinsically bilateral (Chang & Lambon Ralph, 2020).

In Figures 4a and 6e, the parallel traveling waves across the ventral and dorsal visual cortex suggest that reading involves text localization (where) as well as recognition (what), which provides direct evidence for the dual‐stream hypothesis of visual processing (Ungerleider & Haxby, 1994). Furthermore, the spatiotemporal patterns in the two visual pathways were consistent across all tasks involving reading (Figures 4a,c,e and 5a). During a passive listening task, traveling waves emerged from A1 and split into roughly three streams reaching Spt, STS, and ATL in the left hemisphere (Figures 4b,f and 5b). The spatiotemporal patterns in the auditory cortex were similar for all the tasks involving listening to externally delivered speeches (Figures 4b,d,f and 5b,c). The flow patterns within the auditory cortex also provide direct evidence for, at minimum, a dual‐stream model of auditory speech processing (Fridriksson et al., 2016; Hickok & Poeppel, 2007; Rauschecker & Scott, 2009).

During the reading‐aloud task (Figure 4c), the flow patterns in the visual cortex remain consistent with those during silent reading tasks (Figure 4a). Similarly, in the shadowing tasks (Figure 4d), we observed three streams of waves emerging from A1. The consistent and reproducible traveling wave patterns are likely attributable to the underlying functional brain dynamics (Aquino et al., 2012; Chopra et al., 2023; Xu et al., 2023). Compared with unimodal tasks, the extended activations and distinct flow patterns in SMA during simultaneous multimodal tasks (Figure 4c,d) reflect additional recruitment for speech motor preparation (Lima et al., 2016). Activations with the same phases (greenish regions) in premotor and sensorimotor cortices and Spt also reflect motor control and self‐monitoring of speech production (Hickok et al., 2003, 2009; Meister et al., 2007; Pulvermüller et al., 2006).

In the tasks involving both speech perception and production (Figure 4d,f), the phases of activations in the superior temporal cortex were dominated by external stimulation and they were very similar to those in the passive listening tasks (Figure 4b). However, different flow patterns of activations in higher‐level auditory cortex originating from STS were observed during self‐monitoring of speech in tasks involving reading aloud or reciting (Figures 4c,e and 5a). This suggests that reading for speech production and self‐monitoring engages different neural mechanisms than the perception of external speech.

During the simultaneous multimodal tasks, the activation phases of the visual or auditory cortex were less distinguishable from those of the oral sensorimotor cortex (Figure 4c,d). One of the advantages of phase‐encoded designs over nontime‐resolved block designs or event‐related designs is that the delayed reciting in crossmodal tasks not only separates the activation phases of language input (reading or listening) from that of speech output, but also recruits domain‐general cognitive regions involved in working memory and other executive functions (Figure 4e,f). Interestingly, the activation maps for crossmodal tasks seem to indicate that reading‐memorizing‐reciting tasks involve more cognitive effort than listening‐memorizing‐reciting tasks. This reflects functionally distinct mechanisms for translating written to verbal language and temporary buffering of verbal information before speech output prompted by a visual cue. Furthermore, the additional activations in bilateral domain‐general cognitive regions were more prominent in L2 tasks (Figure 4e,f; right panel), suggesting that processing L2 is more cognitively demanding (see Figures 8, 9, 10 and discussion below). Finally, Spt in the left posterior Sylvian fissure displayed phases coherent with the oral and diaphragm sensorimotor areas in the crossmodal tasks (greenish regions in Figure 4e,f). This suggests that Spt is involved in the processing of linguistic information for speech production and monitoring, supporting its role in sensory‐motor integration (Hickok et al., 2003, 2009).

During a reading‐memorizing‐reciting task, traveling waves sequentially propagate through the occipital, parietal, temporal, and prefrontal cortices, and finally reach speech sensorimotor and auditory cortices (Figures 4e, 6c, and 7c). These spatiotemporal flow patterns provide empirical evidence for logistics models of information processing in the brain (Sereno et al., 2022; Zarbakhshnia et al., 2020; Figure 11). Understanding a written sentence may involve “serial assembly of content” operation—similar to scene comprehension from a series of fixations, which mirrors how connected speech is parsed into chunks for analysis in the brain (Giraud & Poeppel, 2012). Individual words are first “packed and carried” from the early visual cortex into ventral and dorsal visual streams by traveling waves through retinotopic areas (Figure 7a,c). The assembled information may then “escape” from the topologically mapped cortex and “spill over” into more domain‐general cognitive regions (e.g., IPL, dlPFC, and rMFG) involved in working memory and other executive functions, while still exhibiting spatially coherent wave‐like activation patterns (Sereno et al., 2022). After brief “storage‐in‐transit” in these regions, the information then flows back into the topologically organized somatomotor cortex to generate speech output, which is simultaneously monitored by the auditory cortex during vocalization. Similar patterns of waves flowing in and out of topologically mapped cortex were observed in the crossmodal and cross‐language tasks (Figures 4 and 5). However, a larger extent of “overspill” into domain‐general cognitive regions (e.g., IPL) was observed in the cross‐language tasks, suggesting that they involved greater cognitive effort.

Moving outside of the topological areas, the surge profile of each sROI provides additional information to compare the arrival, ending, duration, and height of traveling waves within each domain‐general cognitive region across different language tasks (Figures 8, 9, 10). In contrast, regions identified by nontime‐resolved general linear models only show statistically significant activation strength and extent without resolving the temporal information of activations within and between them. In most single‐language tasks, both L1 and L2 exhibited similar activation duration as shown in the surge profiles of the same sROI (e.g., dlPFC). L1 tasks always activated domain‐general cognitive regions earlier than L2 tasks, while L2 tasks induced higher magnitudes, suggesting that L2 processing is indeed more cognitively demanding. During the crossmodal cross‐language processing, domain‐general cognitive regions in L1‐to‐L2 translation tasks triggered activations earlier than in L2‐to‐L1 translation tasks, and L2‐to‐L1 translation tasks induced lower but more extended activation. This suggests that L2‐to‐L1 processing may require lesser but prolonged cognitive effort. Taken together, phase‐encoded fMRI enables the analysis of temporal evolution of activations in domain‐general cognitive regions. The surge profiles of traveling waves can serve as potential indicators of cognitive performances in language and nonlanguage tasks.

## 5 CONCLUSIONS

In this study, we have overcome limitations of existing fMRI techniques, for example, loud gradient‐coil noise and speaking‐induced head motion artifacts (Jolly et al., 2020), and have conducted real‐time fMRI during naturalistic language tasks (reading aloud, listening, shadowing, and consecutive interpreting), unveiling the many facets of human information processing. Maximum efforts were made to enhance ecological validity of the study—besides using natural sentences as stimuli and involving overt speech, we also simulated the real‐world situation of consecutive interpreting by giving a visual cue to prompt the subject's response. Very importantly, we have solved the temporal bottleneck problem of fMRI by using rapid phase‐encoded designs and simultaneously visualized the spatial and temporal unfolding of surface traveling waves across the entire brain during real‐time unimodal, multimodal, crossmodal, and cross‐language tasks. The unfolding of hemodynamic traveling waves, albeit much slower, is strongly reminiscent of neural oscillations (Muller et al., 2018; Wu et al., 2008), suggesting the waves originate from neuronal activities. By analyzing the spatiotemporal traveling wave patterns, we can reveal the real‐time functional connectivity between brain regions activated at each moment (i.e., local and distant activation pattern in each movie frame) as well as hints of the direction of information flows and possible causality between brain regions (as indicated by arrows in Figures 6 and 7 and vector field maps in Figures S5‐S22), thus providing direct evidence for multiple human information processing models in visual, auditory, and sensorimotor cortices (Fridriksson et al., 2016; Hickok & Poeppel, 2007; Huang & Sereno, 2018; Price, 2012; Rauschecker & Scott, 2009; Saura et al., 2008; Ungerleider & Haxby, 1994). We believe that rapid phase‐encoded fMRI can be adapted outside language studies. The ability to track down the step‐by‐step cognitive processes across the whole brain at sub‐second precision may advance our understanding of the computational principles of the widely distributed cognitive network in both normal and abnormal brain functioning.

This study provides a more fine‐grained picture of spatiotemporal information flows in the brain during natural language functioning. To share these results, we have constructed an open access database, the University of Macau Brain Atlas (Movie S19), which incorporates annotated multilayer, multimodal, multilanguage, and multi‐population functional activation maps and movies. Similar to interactive weather websites with dynamic radar maps, UMBA shows the directions of traveling waves (cf. storms paths; Li et al., 2018) during real‐time language processing across the flattened cortical surface. UMBA will undergo continuous updates with new layers of maps and movies from different languages, tasks, and populations, in the hope that it can be used as a “Google Earth‐like” interactive guide by the neuroimaging community (Movie S20).

## AUTHOR CONTRIBUTIONS

*Conceptualization*: VLCL, TIL, DL, and RSH. *Data curation*: TIL, CTL, CUC, and RSH. *Formal analysis*: TIL, LL, CUC, and RSH. *Funding acquisition*: VLCL, MIS, DL, and RSH. *Investigation*: TIL, CTL, CUC, and RSH. *Methodology*: VLCL, TIL, CTL, DL, and RSH. *Project administration*: VLCL, CUC, DL, and RSH. *Resources*: VLCL, MIS, DL, and RSH. *Software*: VLCL, MIS, DL, and RSH. *Supervision*: VLCL, DL, and RSH. *Validation*: RSH. *Visualization*: TIL, LL, and RSH. *Writing—original draft*: VLCL, TIL, and RSH. *Writing—review and editing*: VLCL, TIL, MIS, DL, and RSH.

## FUNDING INFORMATION

This study was supported by University of Macau Development Foundation grant (EXT‐UMDF‐014‐202q1); University of Macau grants (CRG2023‐00016‐FAH, CRG2021‐00001‐ICI, CRG2020‐00001‐ICI, SRG2019‐00189‐ICI, MYRG2022‐00265‐ICI, and MYRG2022‐00200‐FAH); Macau Science and Technology Development Fund (FDCT 0001/2019/ASE); and U.S. National Institutes of Health grant (R01 MH081990 to MIS and RSH).

## CONFLICT OF INTEREST STATEMENT

Authors declare that they have no competing interests.

## Supporting information


**Movie S1.** Traveling waves during a silent reading task in L1.

**Movie S2.** Traveling waves during a listening task in L1.

**Movie S3.** Traveling waves during a reading‐aloud task in L1.

**Movie S4.** Traveling waves during a shadowing task in L1.

**Movie S5.** Traveling waves during a reading‐memorizing‐reciting task in L1.

**Movie S6.** Traveling waves during a listening‐memorizing‐reciting task in L1.

**Movie S7.** Traveling waves during a silent reading task in L2.

**Movie S8.** Traveling waves during a listening task in L2.

**Movie S9.** Traveling waves during a reading‐aloud task in L2.

**Movie S10.** Traveling waves during a shadowing task in L2.

**Movie S11.** Traveling waves during a reading‐memorizing‐reciting task in L2.

**Movie S12.** Traveling waves during a listening‐memorizing‐reciting task in L2.

**Movie S13.** Traveling waves during an L1‐to‐L2 sight interpreting task.

**Movie S14.** Traveling waves during an L1‐to‐L2 consecutive speech interpreting task.

**Movie S15.** Traveling waves during an L1‐to‐L2 consecutive digit interpreting task.

**Movie S16.** Traveling waves during an L2‐to‐L1 sight interpreting task.

**Movie S17.** Traveling waves during an L2‐to‐L1 consecutive speech interpreting task.

**Movie S18.** Traveling waves during an L2‐to‐L1 consecutive digit interpreting task.

**Movie S19.** A snapshot of the University of Macau Brain Atlas (UMBA) website.

**Movie S20.** Tracking “brainstorms” on an earth‐like surface. The group‐average activation map of the Chinese reading‐memorizing‐reciting task (see Figure 4e, left) is displayed on the spherical cortical surface of the left hemisphere of a single subject. Color contours represent the borders of topological areas (Sereno et al., 2022). Moving white strips represent traveling waves.

**Data S1.** Supporting information.

## Data Availability

The University of Macau Brain Atlas is available at https://cstic.um.edu.mo/umba. Custom codes for analyzing phase‐encoded fMRI data and traveling wave visualization are included in csurf (a FreeSurfer‐compatible package) available for download at https://pages.ucsd.edu/~msereno/csurf/ or https://mri.sdsu.edu/sereno/csurf/. The data that support the findings of this study are available from the corresponding author upon reasonable request.
