# Supplementary material for: Phase‐encoded fMRI tracks down brainstorms of natural language processing with subsecond precision
Source: Hum Brain Mapp. 2024 Feb 9;45(2):e26617. doi: 10.1002/hbm.26617 (PMC10858339; doi:10.1002/hbm.26617)
Supplement: Supplementary file 21 — Data S1. Supporting information. [file HBM-45-e26617-s019.pdf]

Supporting Information

Phase-encoded fMRI tracks down brainstorms of natural language processing with sub-second precision

Victoria Lai Cheng Lei[1,2†], Teng Ieng Leong[1,2†], Cheok Teng Leong[1,3], Lili Liu[1,3], Chi Un Choi[1], Martin I. Sereno[4], Defeng Li[1,2*], Ruey-Song Huang[1,3*]

[1]Centre for Cognitive and Brain Sciences, University of Macau, Taipa, Macau SAR, China
[2]Faculty of Arts and Humanities, University of Macau, Taipa, Macau SAR, China
[3]Faculty of Science and Technology, University of Macau, Taipa, Macau SAR, China
[4]Department of Psychology, San Diego State University, San Diego, CA, United States

[†]These authors contributed equally to this work

*Corresponding authors: Ruey-Song Huang, Defeng Li
Email: rshuang@um.edu.mo, defengli@um.edu.mo

This PDF file includes:

Figures S1 to S22

Tables S1 to S3

Legends for Movies S1 to S20

Other supporting materials for this manuscript include the following:

Movies S1 to S20

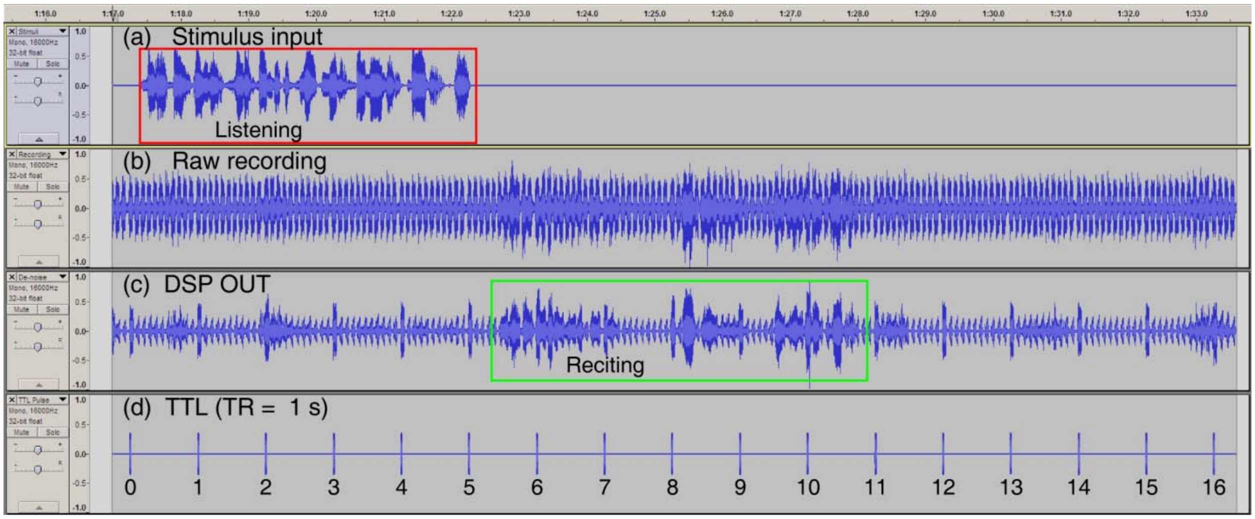

Figure S1. Audio recording. Sample soundtracks of a single trial in an L2 listening-memorizing-reciting task. (a) Recording of audio stimulus input. (b) Raw recording: speech output recorded with unprocessed MRI noise. (c) Digital signal processing (DSP) OUT: audio output after applying real-time noise cancellation to the raw recording in (b). (d) Transistor–transistor logic (TTL) pulse: timestamps recorded at the beginning of each TR. The screenshot of recordings was created using Audacity® (Audacity® software is copyright © 1999-2021 Audacity Team. The name Audacity® is a registered trademark. https://www.audacityteam.org/about/citations-screenshots-and-permissions/).

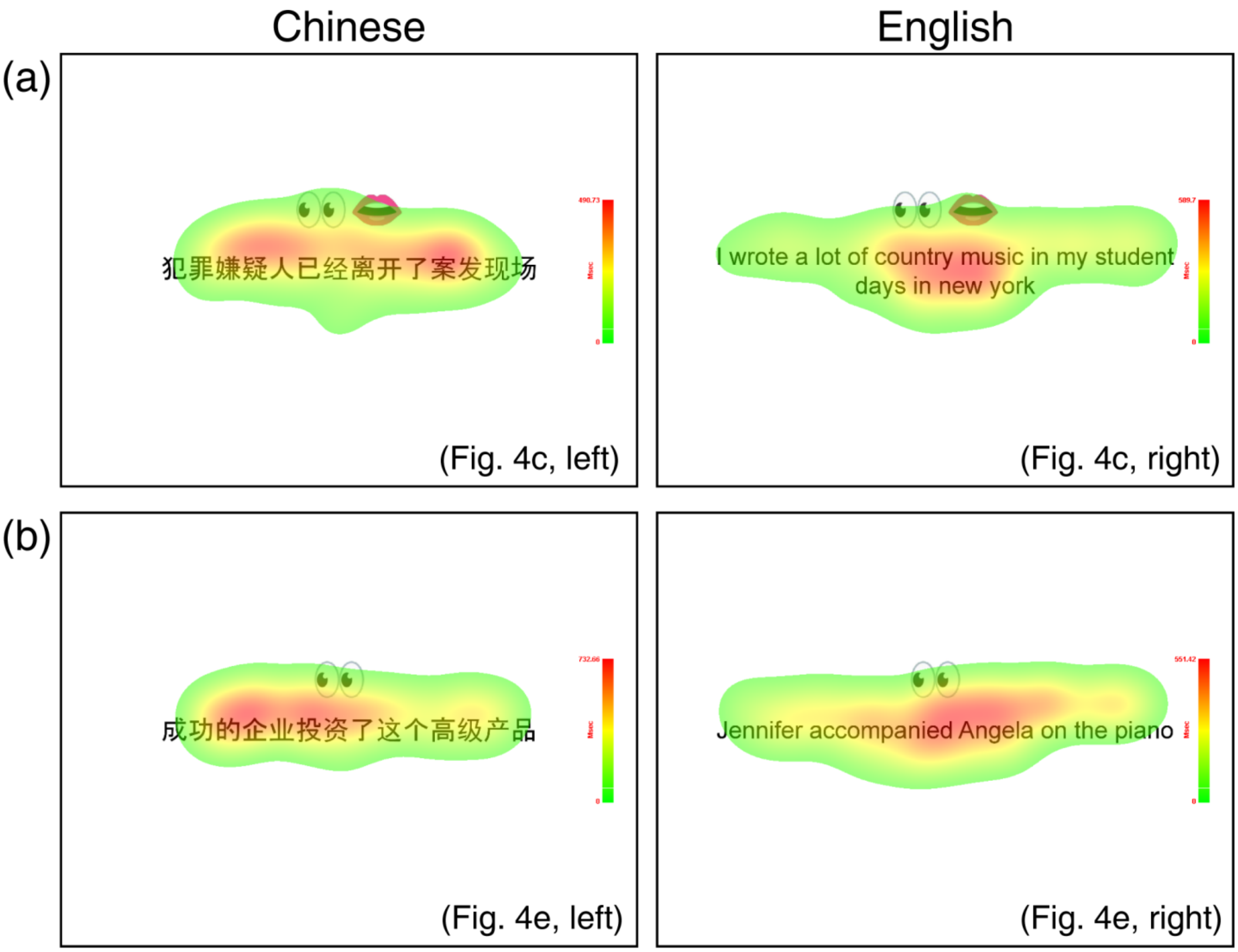

Figure S2. Eye-tracking heat maps. Results of eye-tracking for tasks involving reading in one subject. (a) Heat maps for reading-aloud tasks (see Figure 4c). (b) Heat maps for reading-memorizing-reciting tasks (see Figure 4e). (Left panels) Chinese, (Right panels) English. English translation of Chinese sentences presented in the left panels: (a) *The suspect has already left the crime scene*, and (b) *Successful enterprises invested on this premium product*.

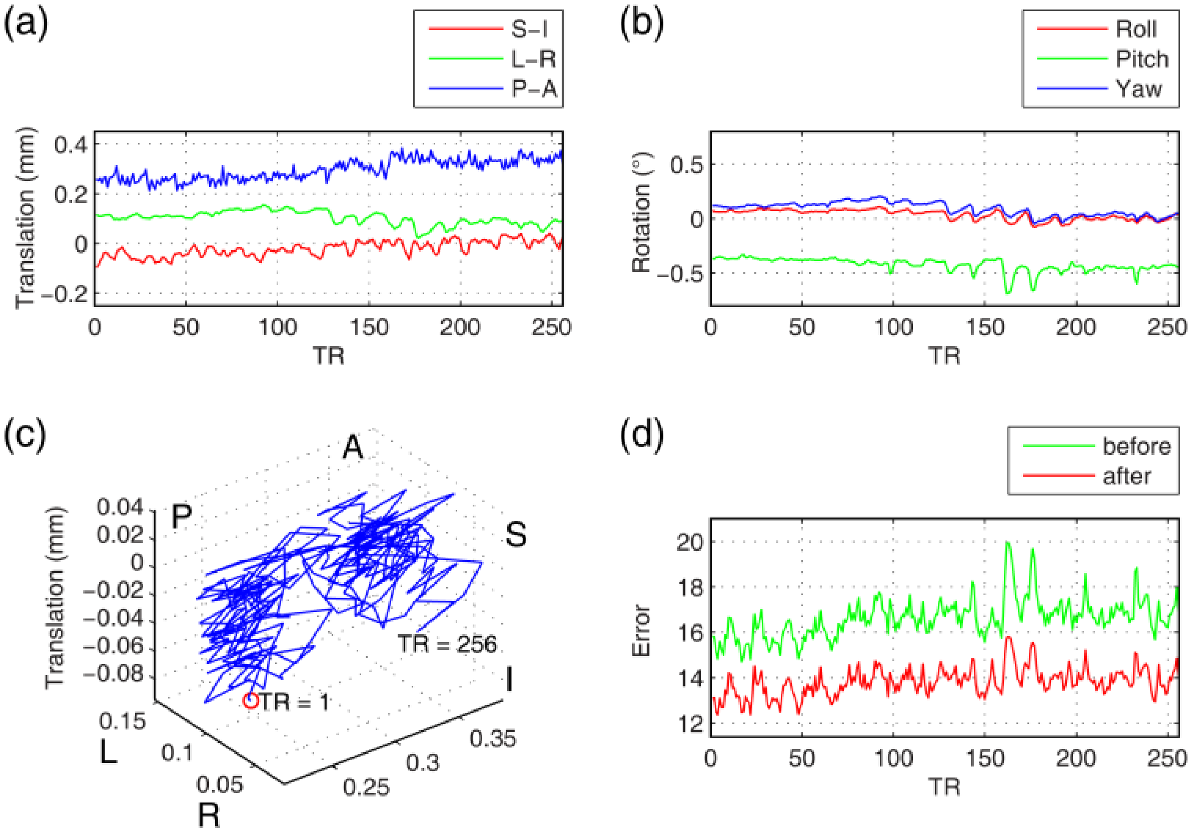

Figure S3. Head motion trajectory. Results of head motion correction for a single scan involving speaking. (a) Head translation (mm) in three axes: superior-inferior (S-I), left-right (L-R), and posterior-anterior (P-A) directions. (b) Head rotation (degree) in three directions. (c) 3D trajectory of head motion from TR=1 to TR=256. (d) Time series of root-mean-square difference (error) between each volume and the reference volume, before and after motion correction.

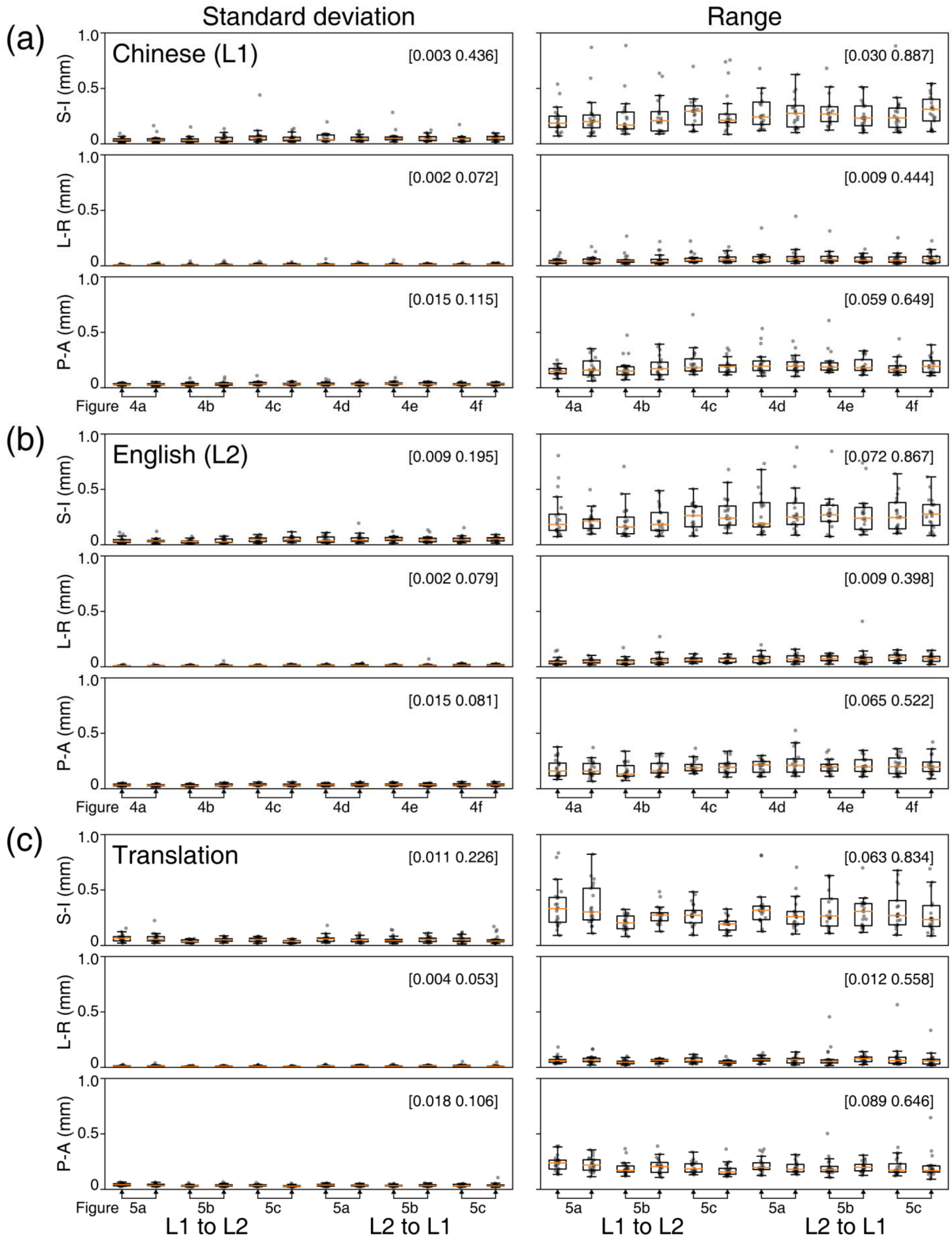

Standard deviation
Range
(a)
Chinese (L1)
[0.003 0.436]
[0.030 0.887]
[0.002 0.072]
[0.009 0.444]
[0.015 0.115]
[0.059 0.649]
S-I (mm)
L-R (mm)
P-A (mm)
Figure 4a 4b 4c 4d 4e 4f
(b)
English (L2)
[0.009 0.195]
[0.072 0.867]
[0.002 0.079]
[0.009 0.398]
[0.015 0.081]
[0.065 0.522]
(c)
Translation
[0.011 0.226]
[0.063 0.834]
[0.004 0.053]
[0.012 0.558]
[0.018 0.106]
[0.089 0.646]
Figure 5a 5b 5c 5a 5b 5c
L1 to L2
L2 to L1

Figure S4. Head motion statistics across subjects. Standard deviation and range (max – min) of head motion (mm) across subjects and tasks in three directions: superior-inferior (S-I), left-right (L-R) and posterior-anterior (P-A). (a) Head motion statistics during Chinese (L1) tasks. (b) Head motion statistics during English (L2) tasks. (c) Head motion statistics during translation tasks. Each boxplot corresponds to a task in Figures 4 and 5 as labelled. Square brackets denote the minimum and maximum values across subjects and tasks.

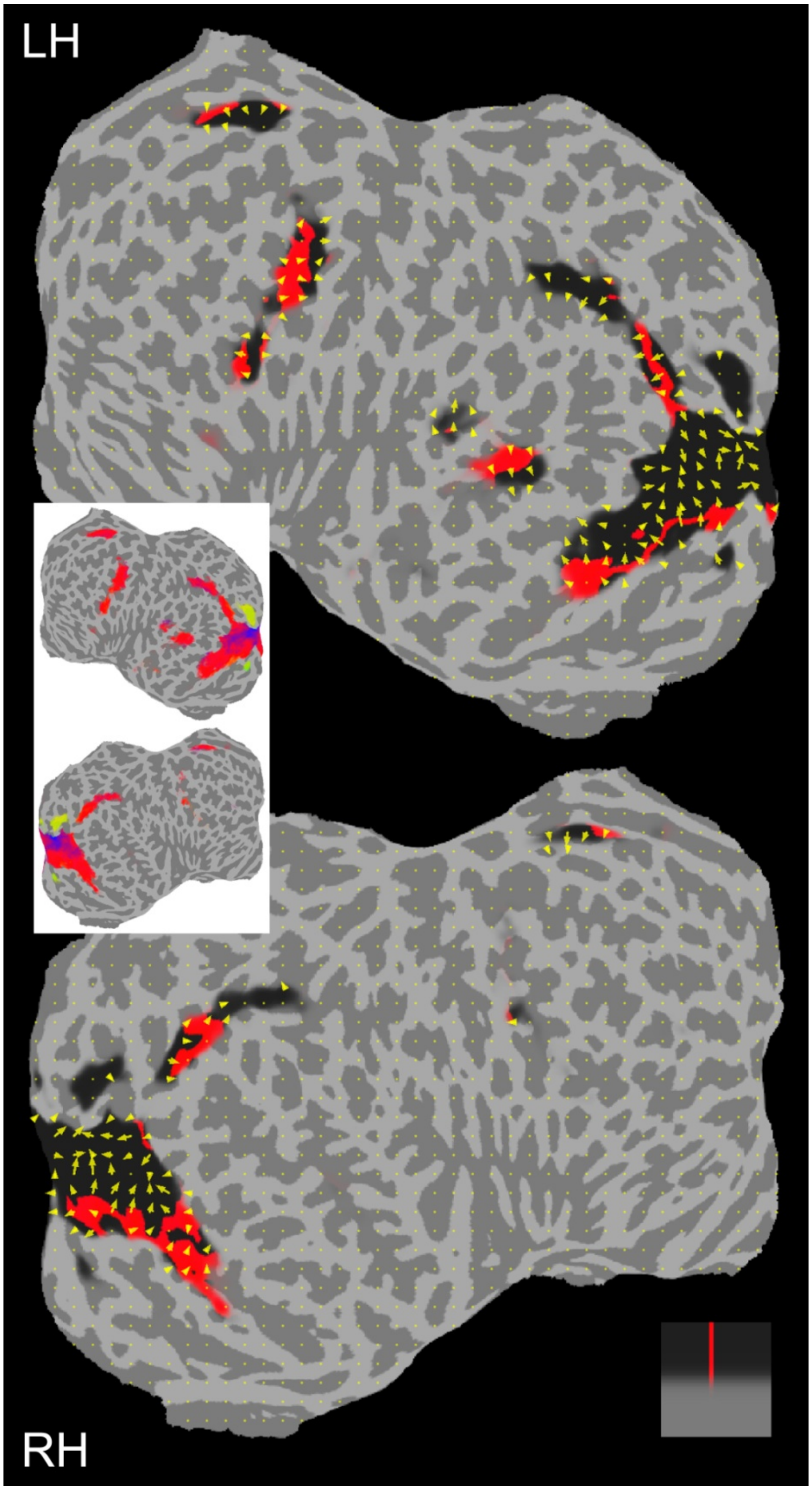

Figure S5. Group-average traveling waves with vector field overlay of the Chinese (L1) reading task. Main figure corresponds to Movie S1. Inset corresponds to Figure 4a, left. LH: left hemisphere; RH: right hemisphere.

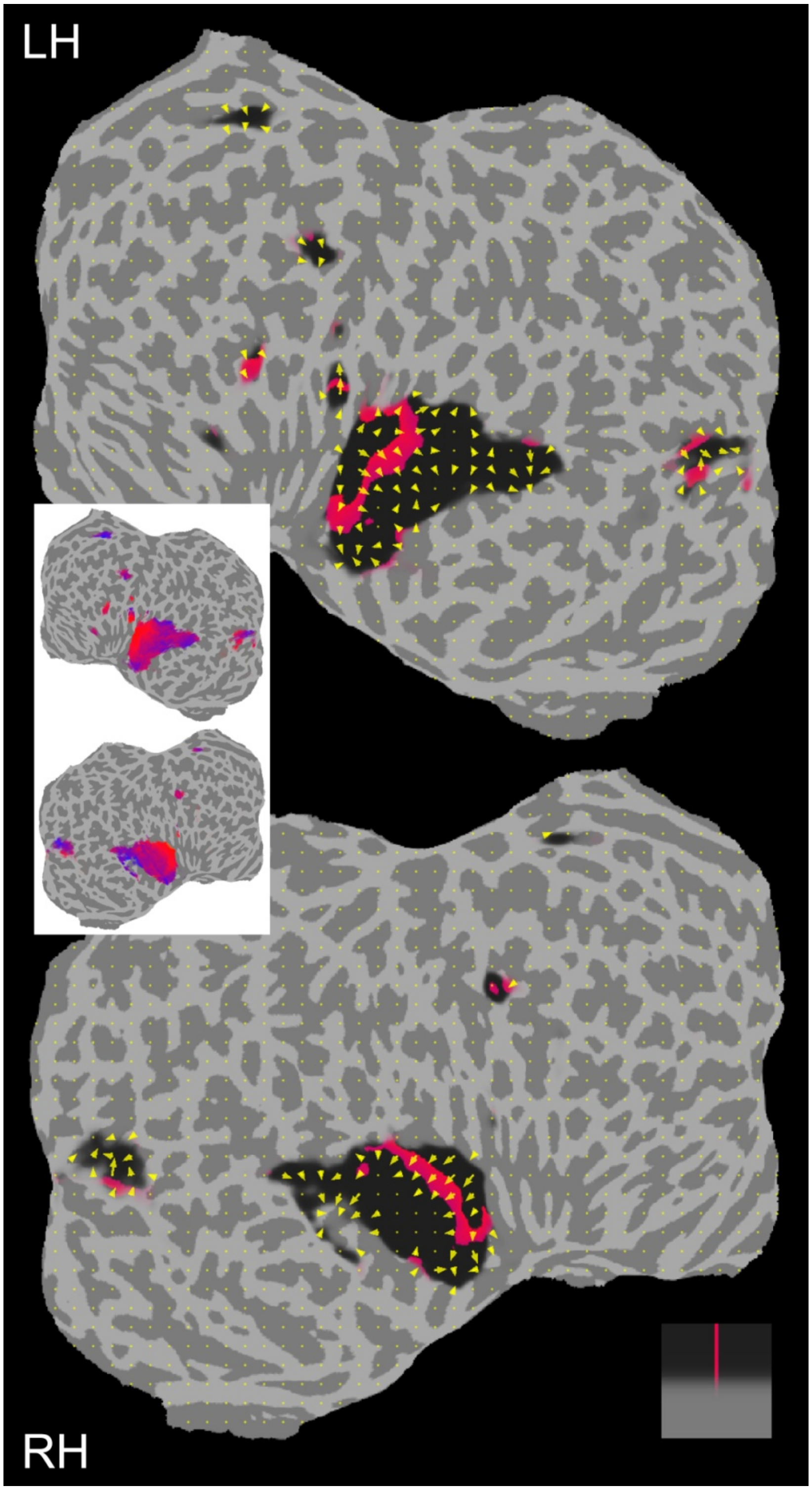

Figure S6. Group-average traveling waves with vector field overlay of the Chinese (L1) listening task. Main figure corresponds to Movie S2. Inset corresponds to Figure 4b, left. LH: left hemisphere; RH: right hemisphere.

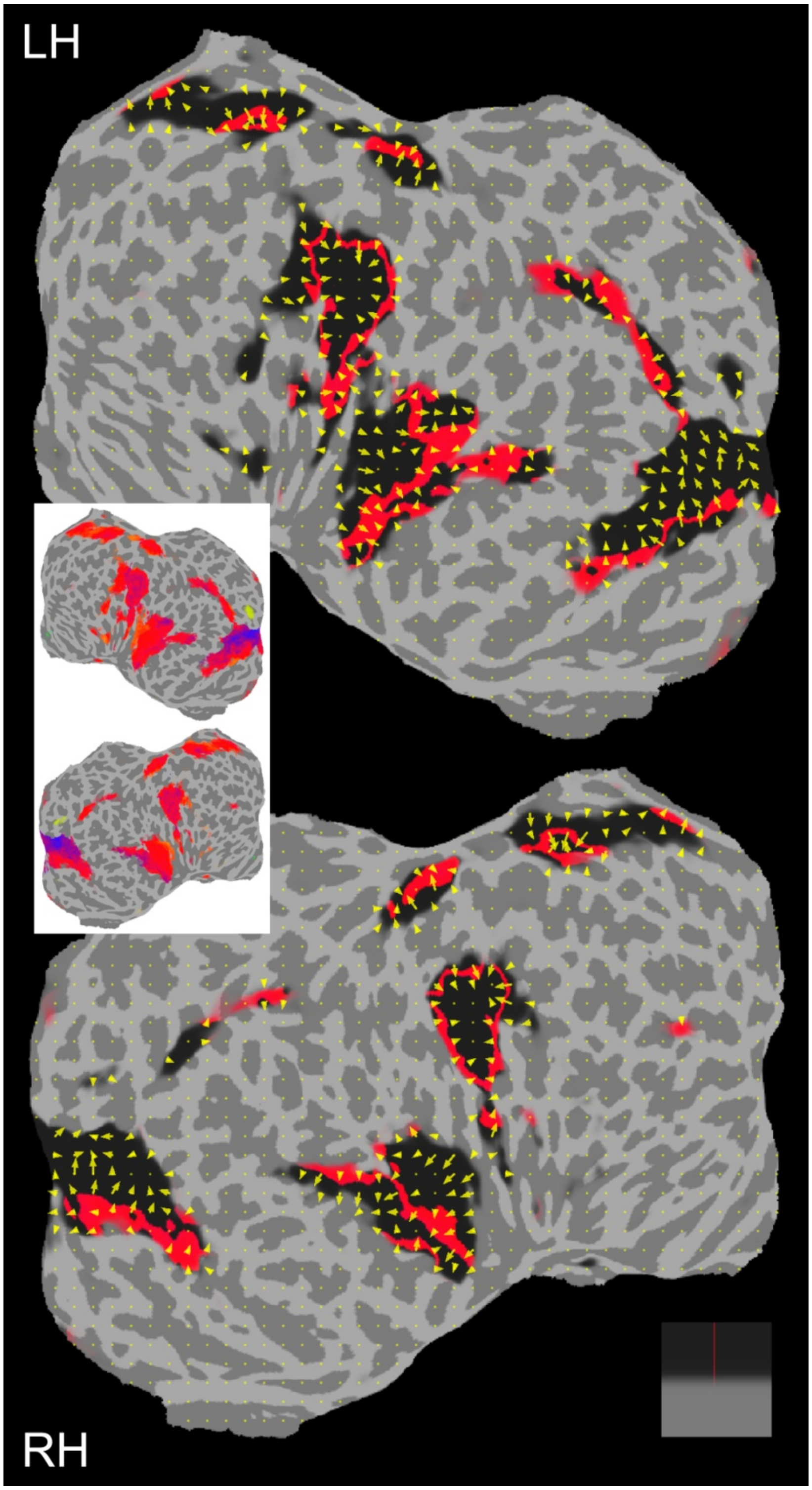

Figure S7. Group-average traveling waves with vector field overlay of the Chinese (L1) reading-aloud task. Main figure corresponds to Movie S3. Inset corresponds to Figure 4c, left. LH: left hemisphere; RH: right hemisphere.

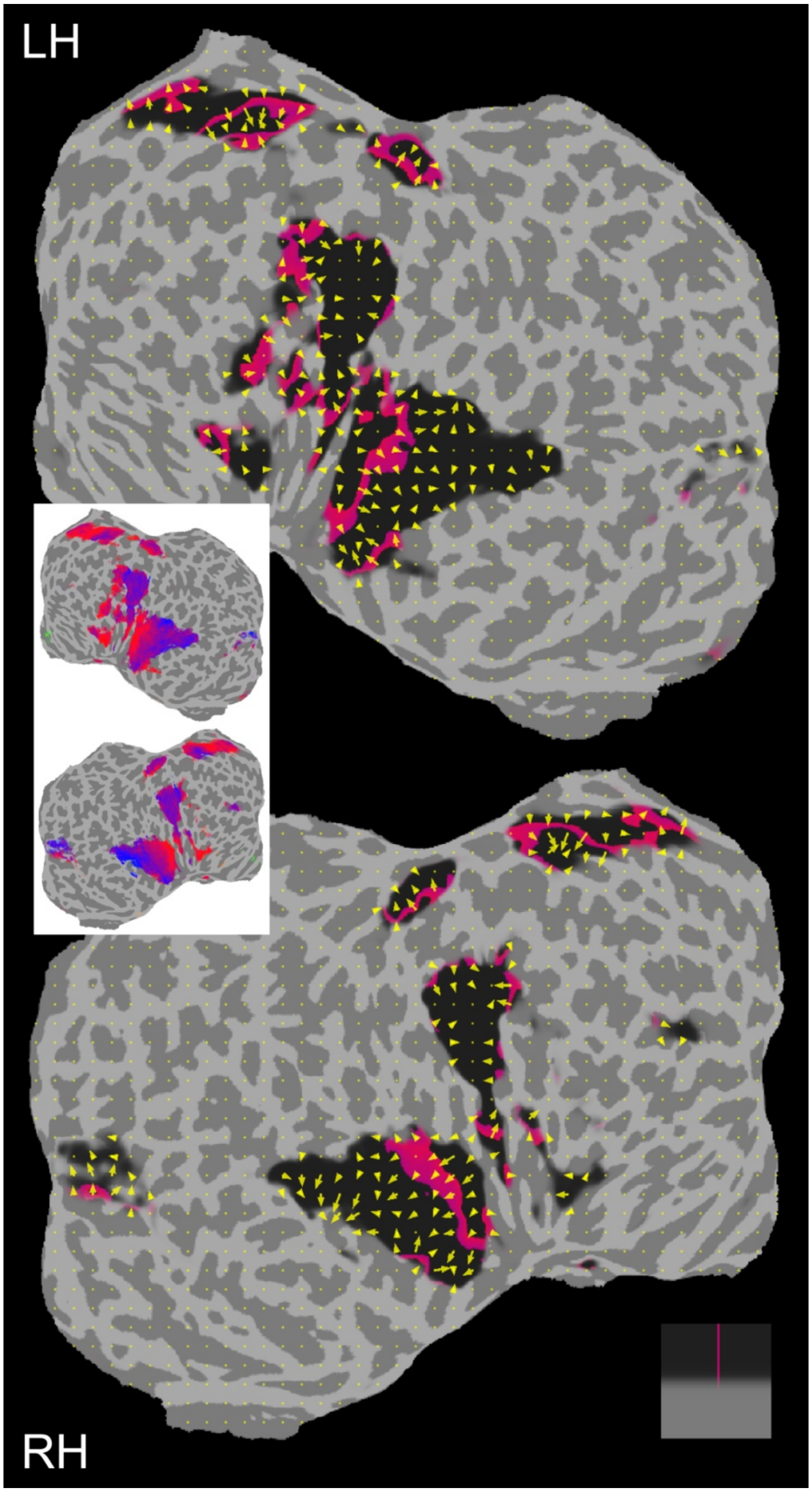

Figure S8. Group-average traveling waves with vector field overlay of the Chinese (L1) shadowing task. Main figure corresponds to Movie S4. Inset corresponds to Figure 4d, left. LH: left hemisphere; RH: right hemisphere.

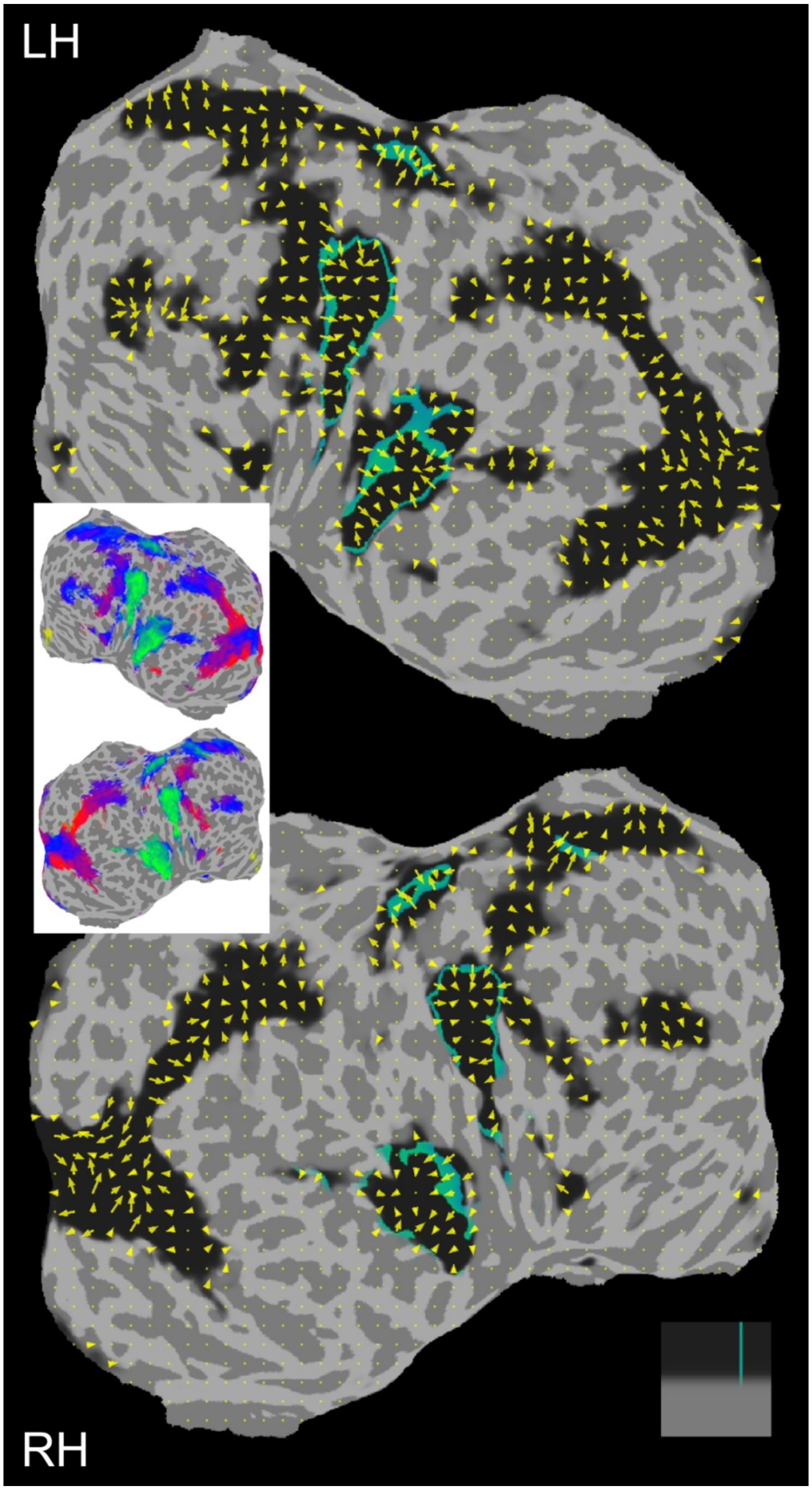

Figure S9. Group-average traveling waves with vector field overlay of the Chinese (L1) reading-memorizing-reciting task. Main figure corresponds to Movie S5. Inset corresponds to Figure 4e, left. LH: left hemisphere; RH: right hemisphere.

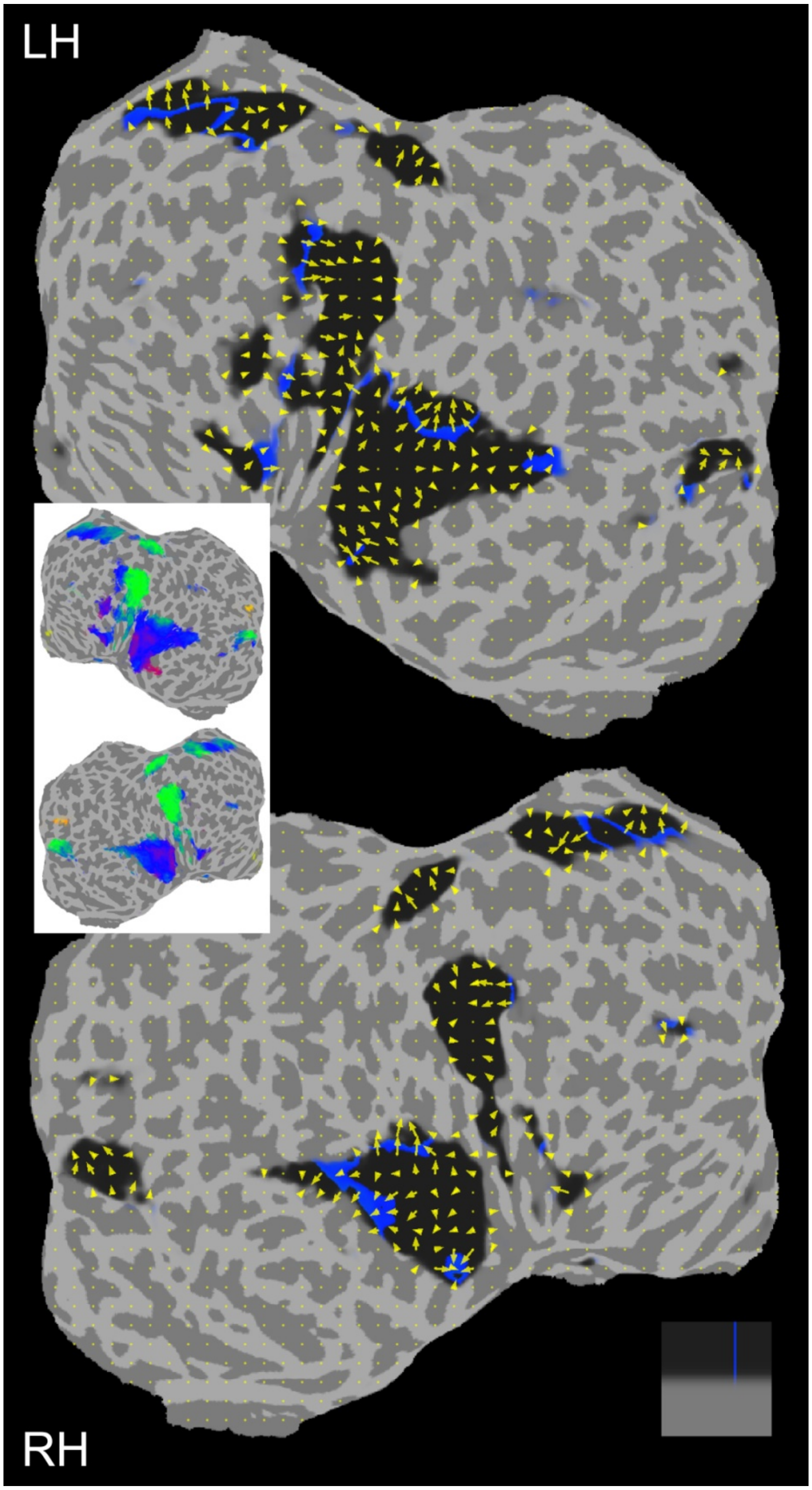

Figure S10. Group-average traveling waves with vector field overlay of the Chinese (L1) listening-memorizing-reciting task. Main figure corresponds to Movie S6. Inset corresponds to Figure 4f, left. LH: left hemisphere; RH: right hemisphere.

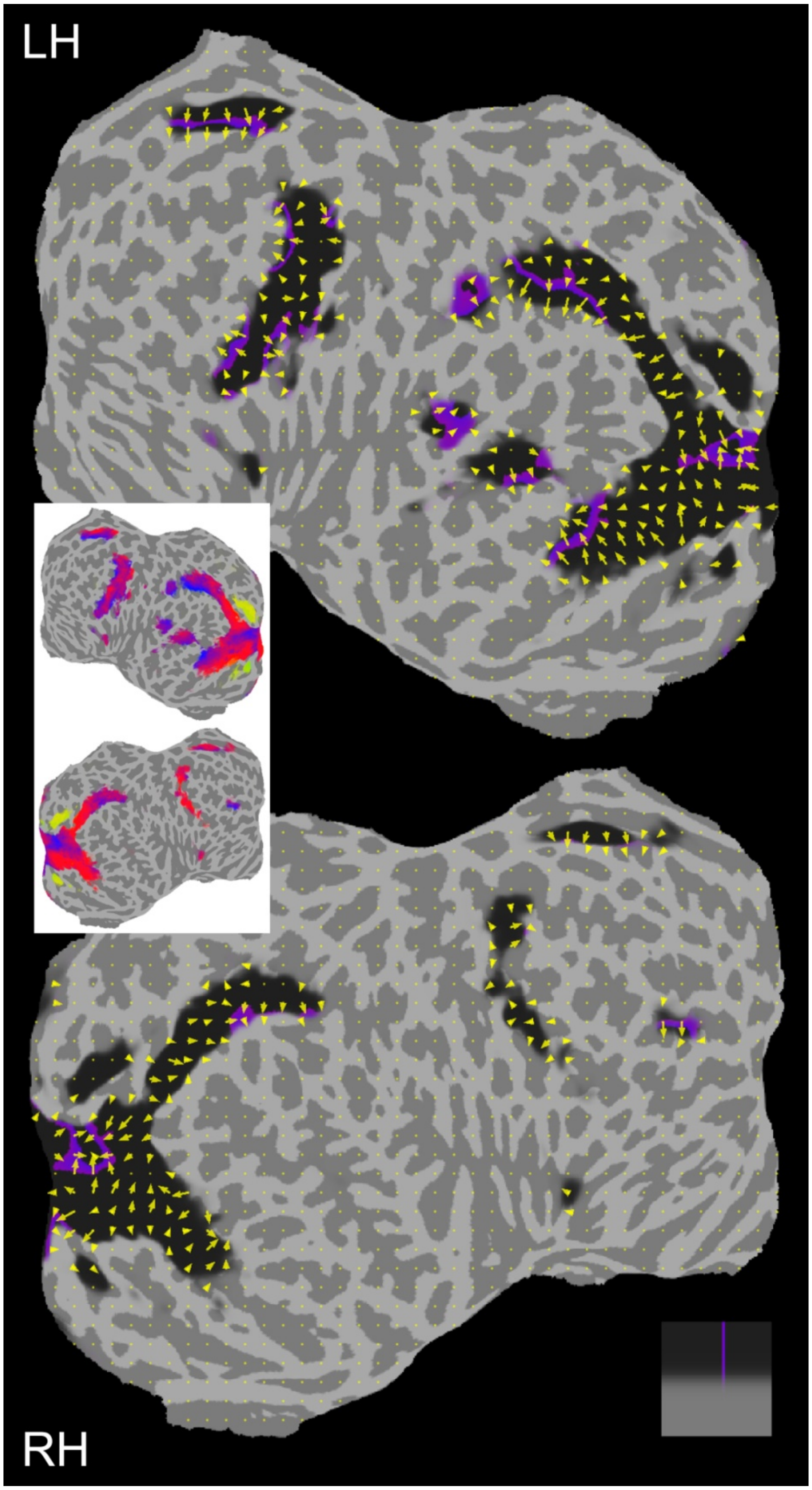

Figure S11. Group-average traveling waves with vector field overlay of the English (L2) reading task. Main figure corresponds to Movie S7. Inset corresponds to Figure 4a, right. LH: left hemisphere; RH: right hemisphere.

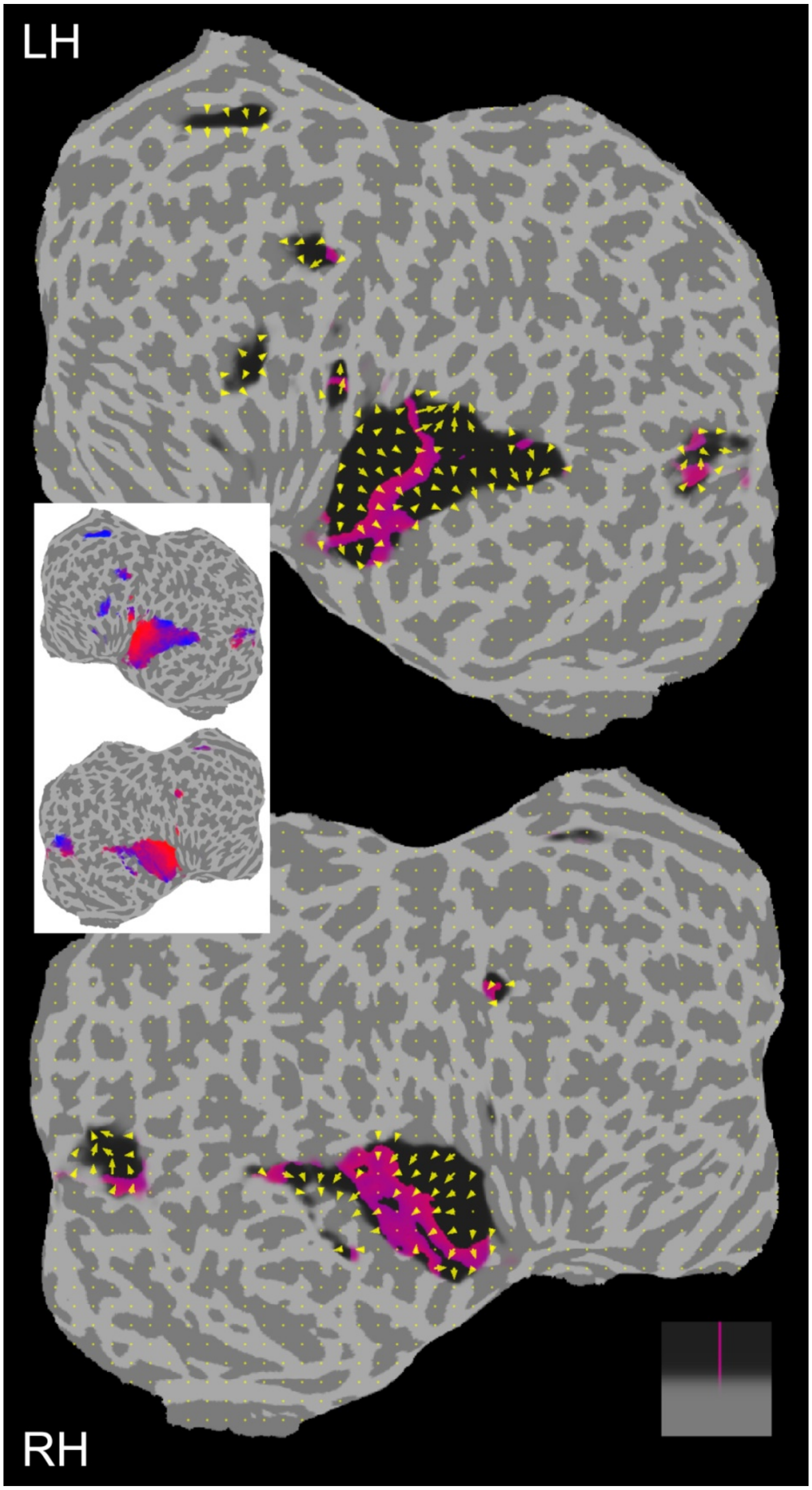

Figure S12. Group-average traveling waves with vector field overlay of the English (L2) listening task. Main figure corresponds to Movie S8. Inset corresponds to Figure 4b, right. LH: left hemisphere; RH: right hemisphere.

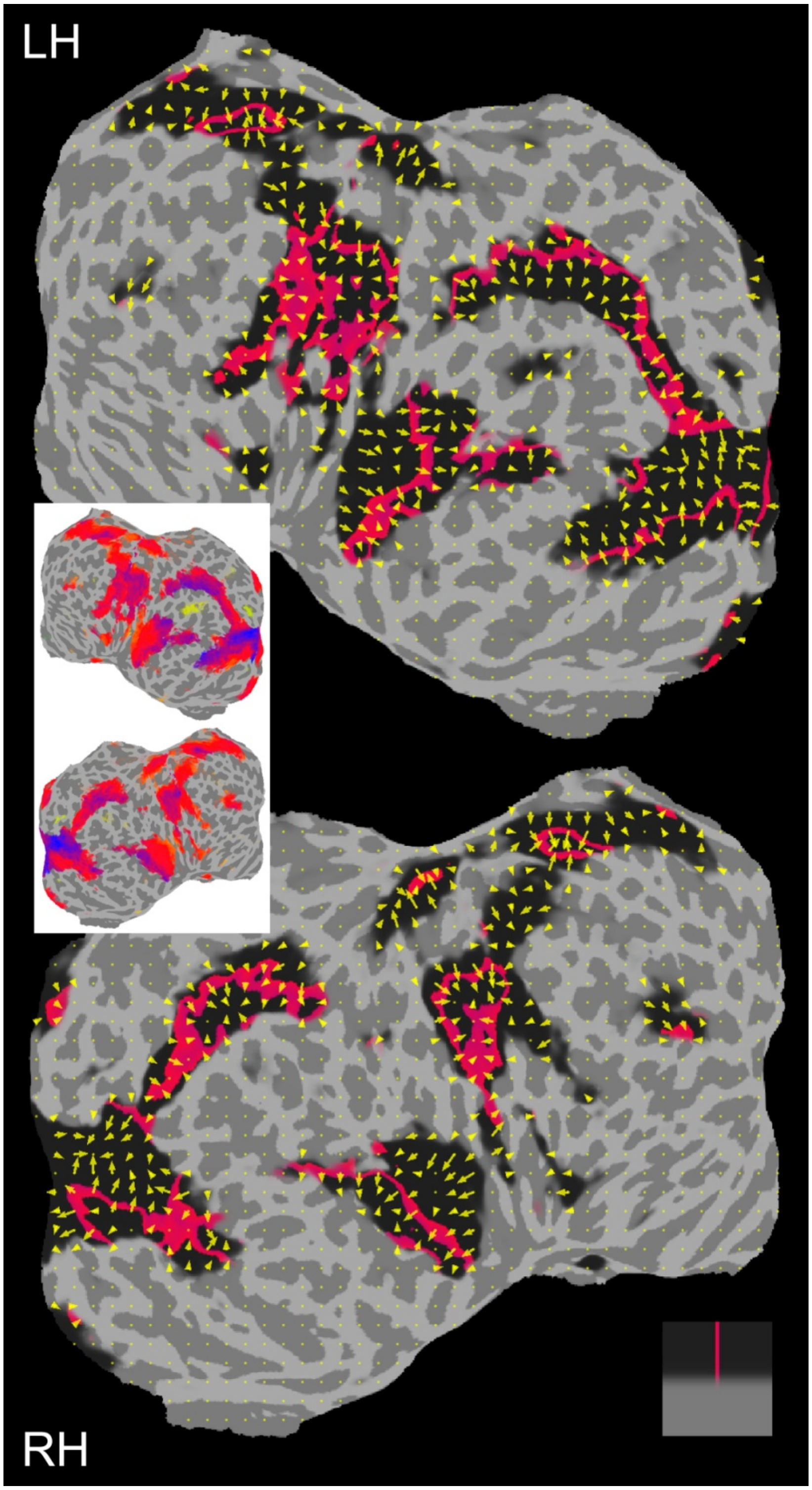

Figure S13. Group-average traveling waves with vector field overlay of the English (L2) reading-aloud task. Main figure corresponds to Movie S9. Inset corresponds to Figure 4c, right. LH: left hemisphere; RH: right hemisphere.

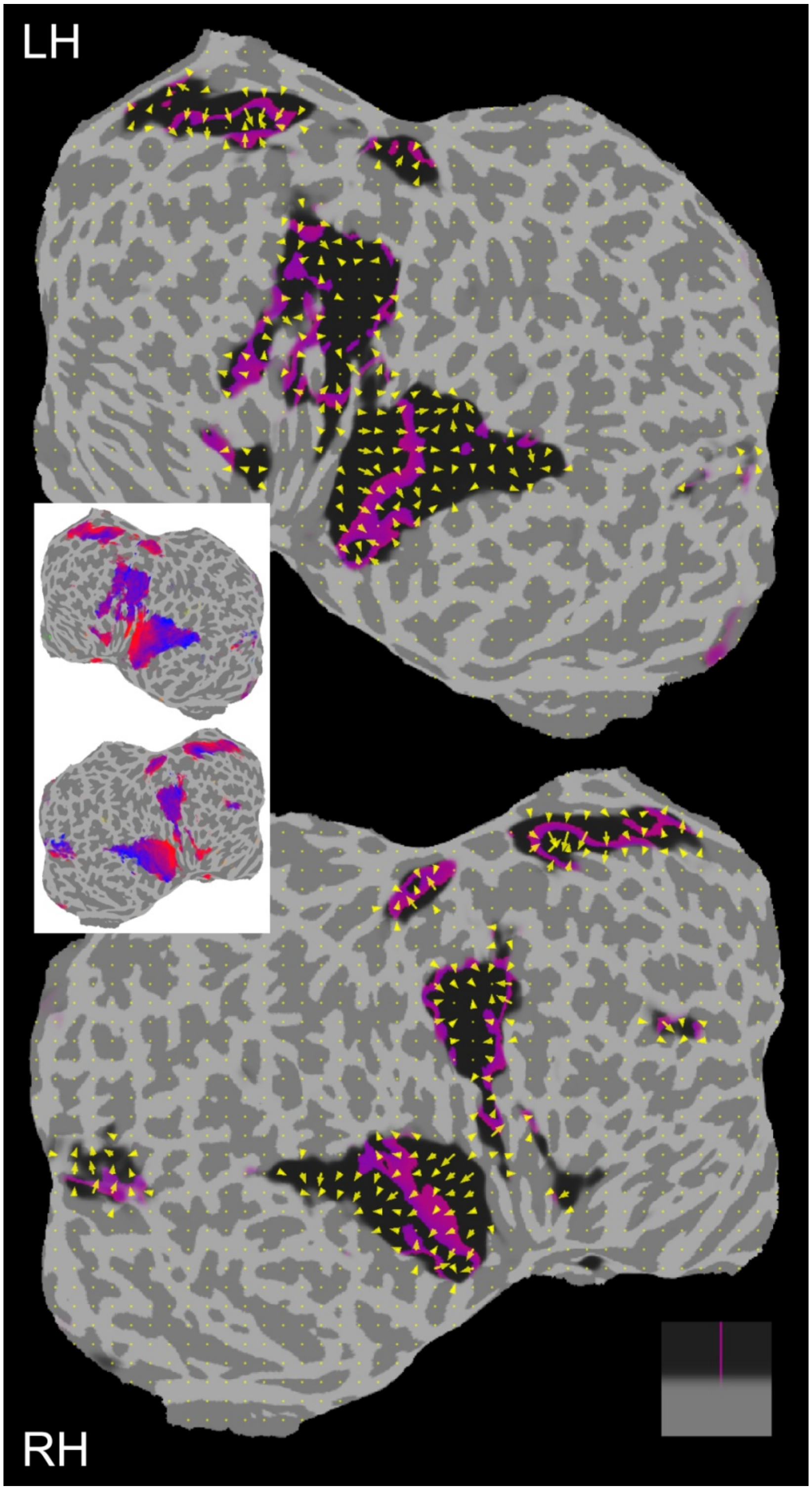

Figure S14. Group-average traveling waves with vector field overlay of the English (L2) shadowing task. Main figure corresponds to Movie S10. Inset corresponds to Figure 4d, right. LH: left hemisphere; RH: right hemisphere.

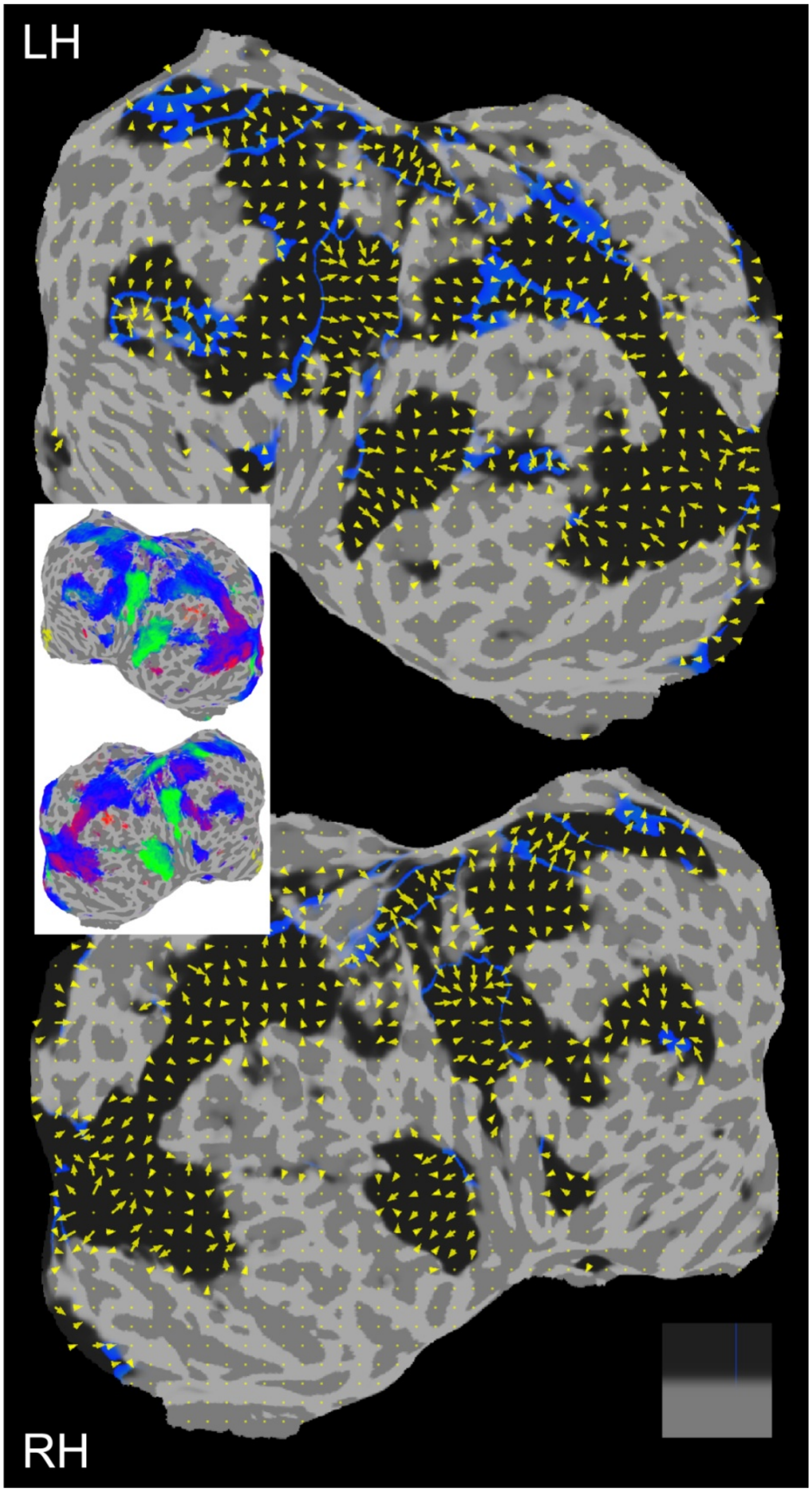

Figure S15. Group-average traveling waves with vector field overlay of the English (L2) reading-memorizing-reciting task. Main figure corresponds to Movie S11. Inset corresponds to Figure 4e, right. LH: left hemisphere; RH: right hemisphere.

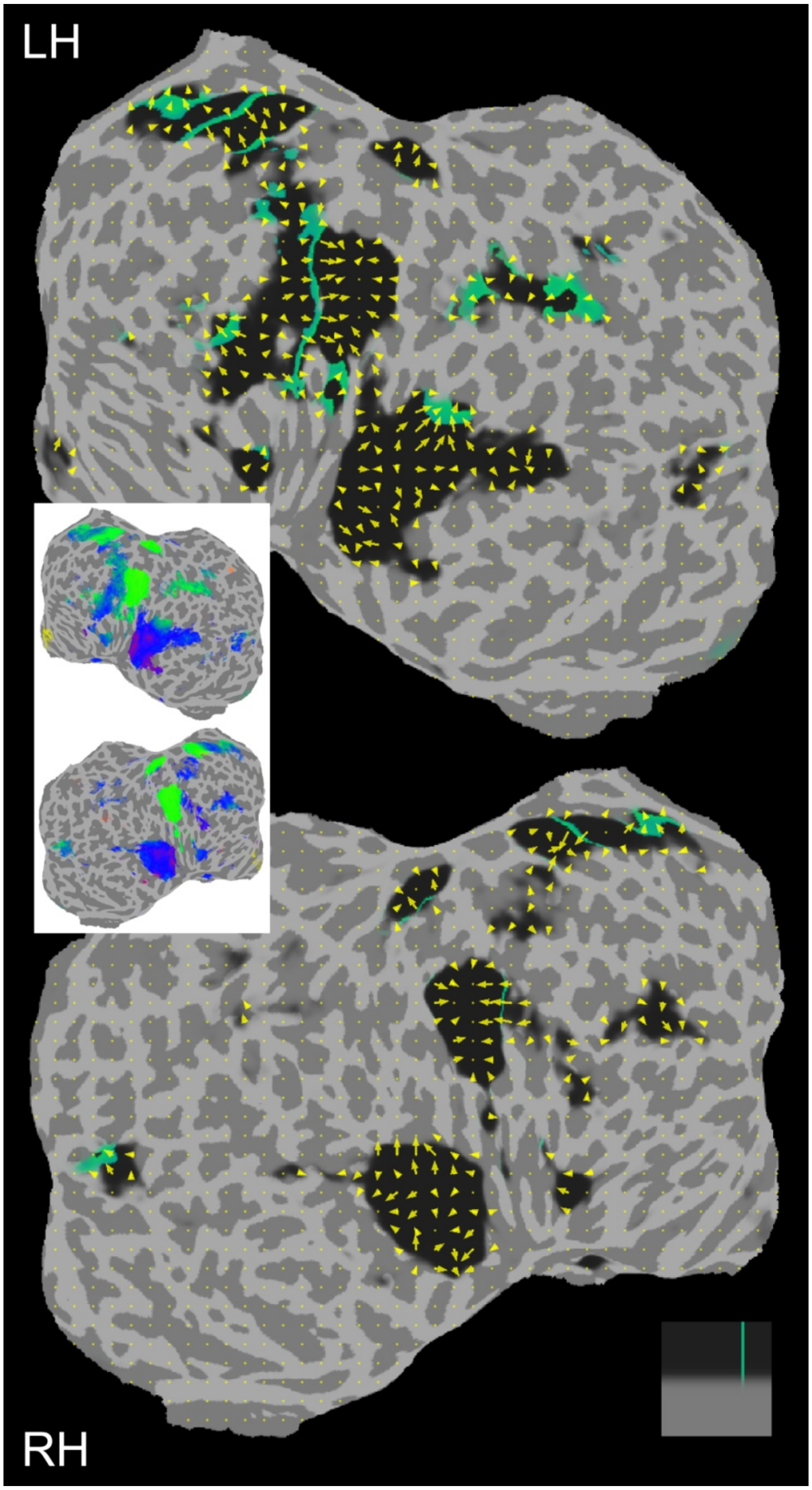

Figure S16. Group-average traveling waves with vector field overlay of the English (L2) listening-memorizing-reciting task. Main figure corresponds to Movie S12. Inset corresponds to Figure 4f, right. LH: left hemisphere; RH: right hemisphere.

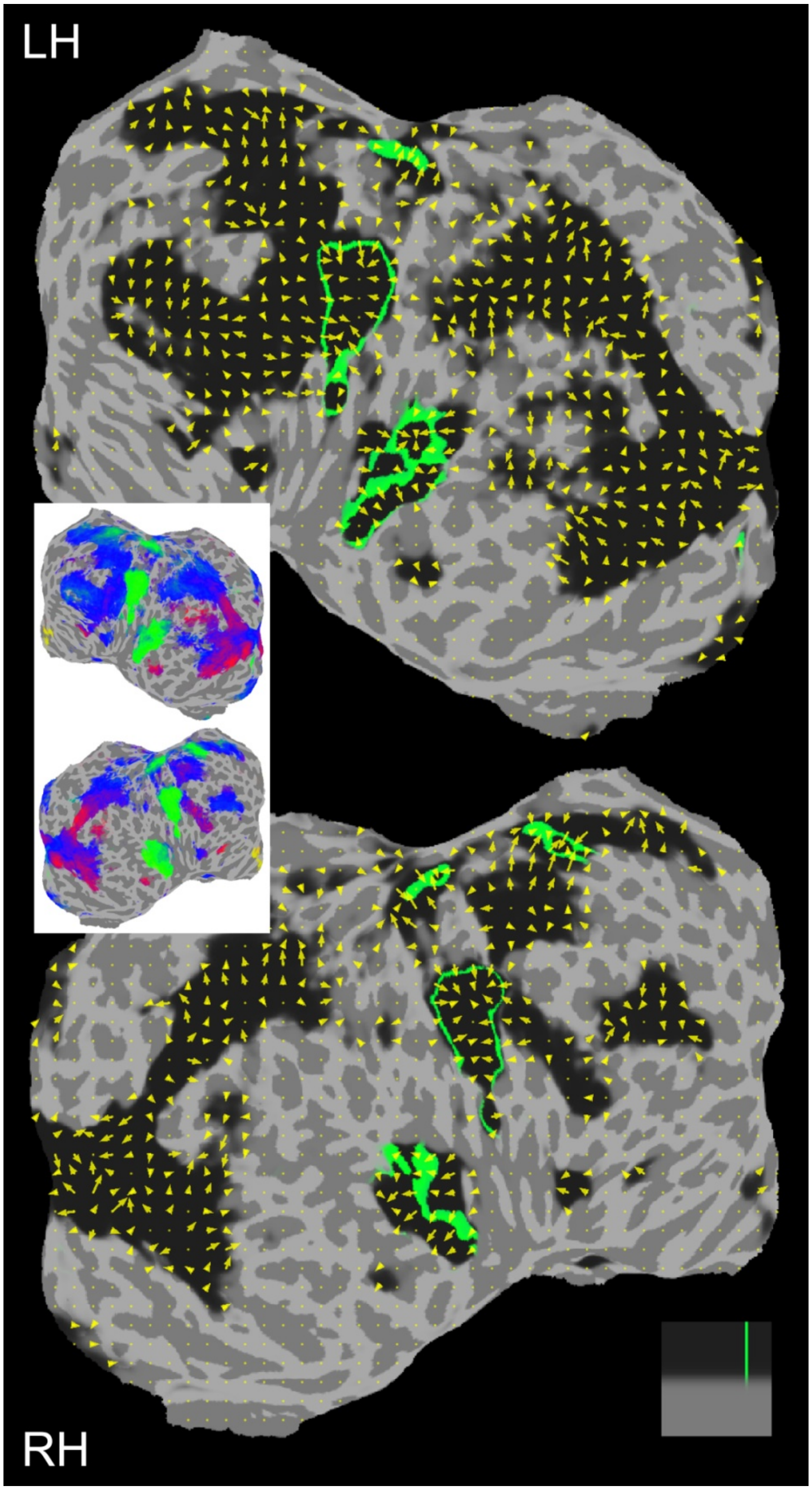

Figure S17. Group-average traveling waves with vector field overlay of the Chinese (L1) to English (L2) reading-memorizing-translating task. Main figure corresponds to Movie S13. Inset corresponds to Figure 5a, left. LH: left hemisphere; RH: right hemisphere.

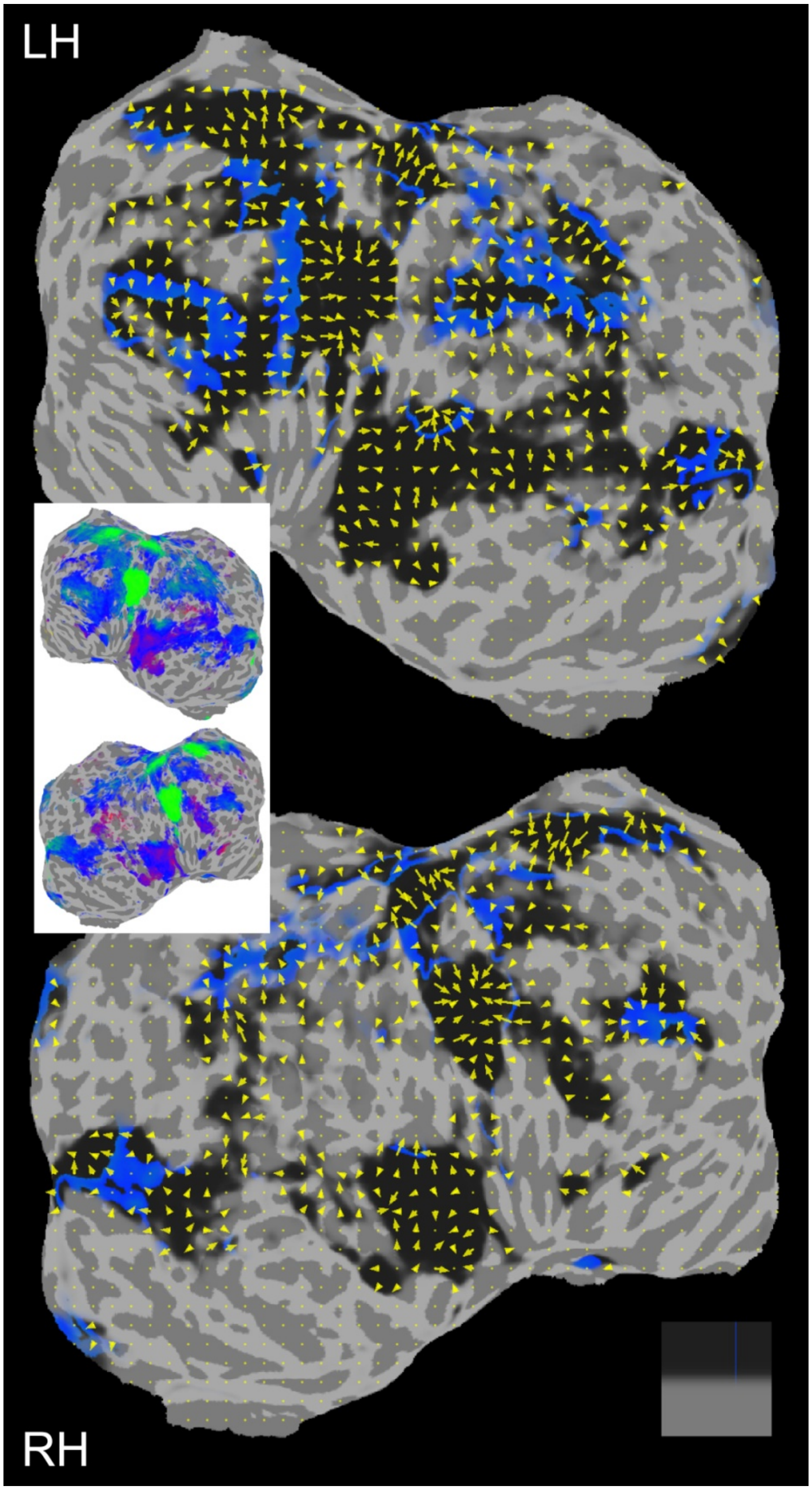

Figure S18. Group-average traveling waves with vector field overlay of the Chinese (L1) to English (L2) listening-memorizing-translating task. Main figure corresponds to Movie S14. Inset corresponds to Figure 5b, left. LH: left hemisphere; RH: right hemisphere.

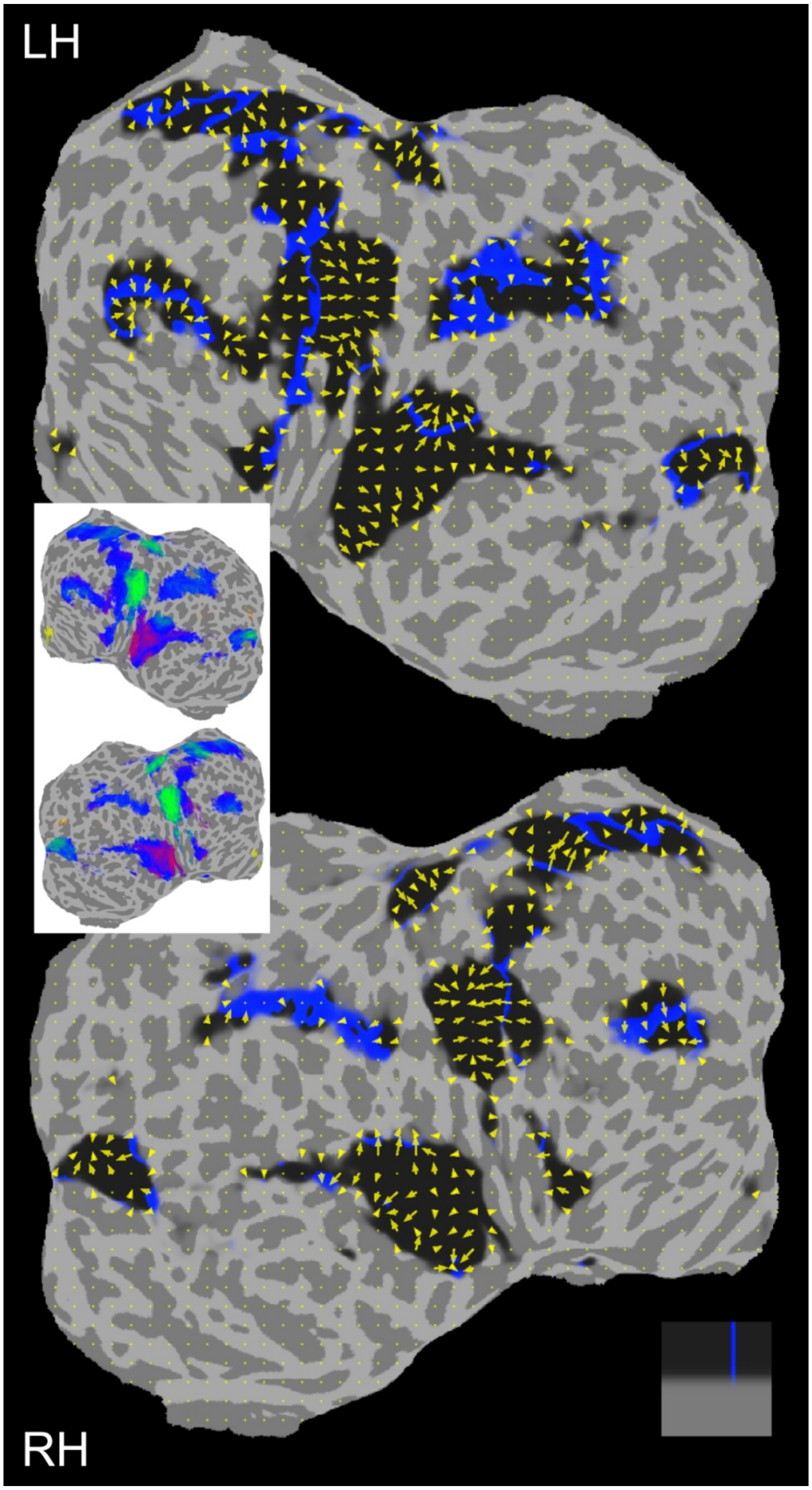

Figure S19. Group-average traveling waves with vector field overlay of the Chinese (L1) to English (L2) listening-memorizing-translating digits task. Main figure corresponds to Movie S15. Inset corresponds to Figure 5c, left. LH: left hemisphere; RH: right hemisphere.

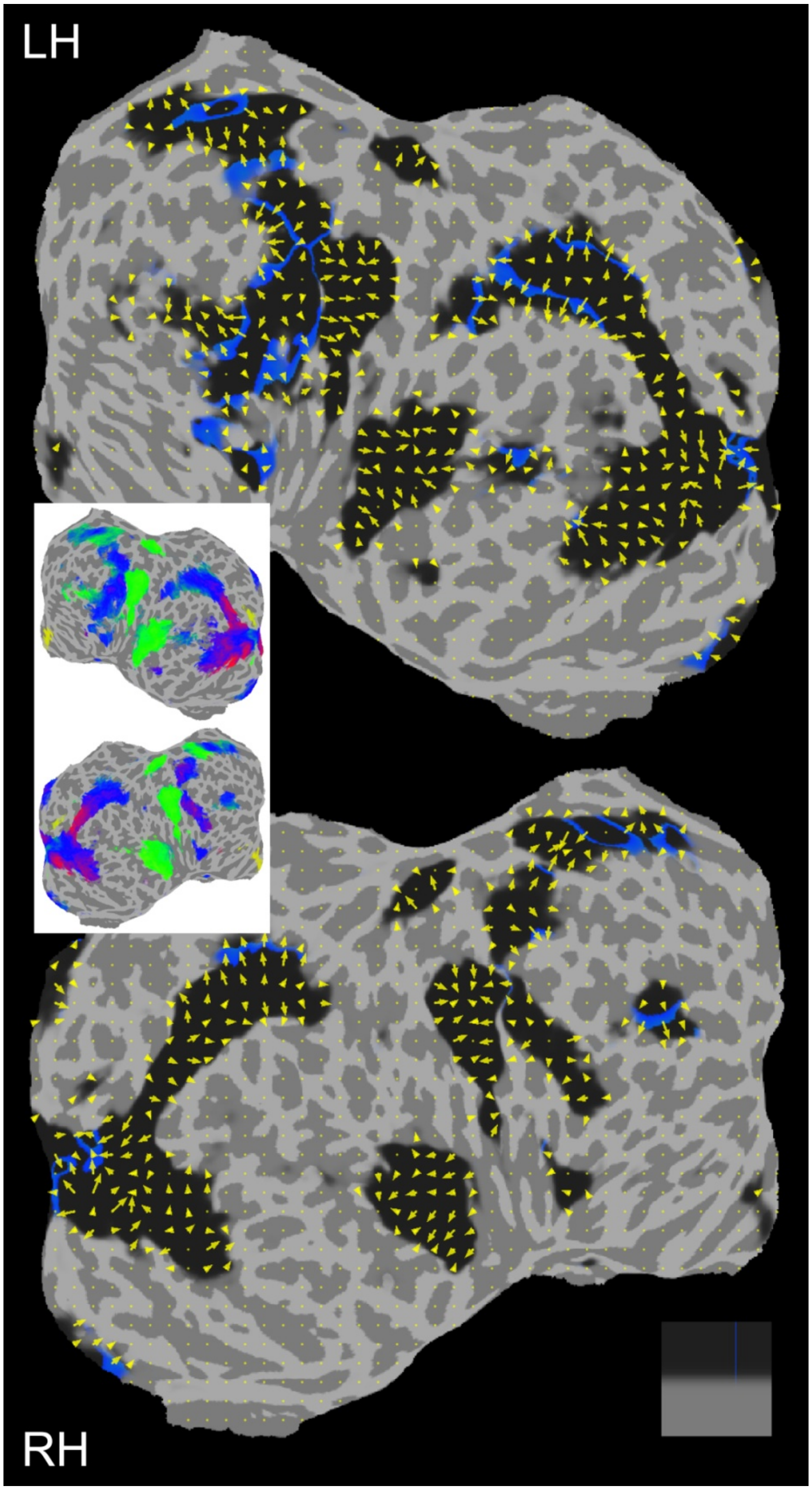

Figure S20. Group-average traveling waves with vector field overlay of the English (L2) to Chinese (L1) reading-memorizing-translating task. Main figure corresponds to Movie S16. Inset corresponds to Figure 5a, right. LH: left hemisphere; RH: right hemisphere.

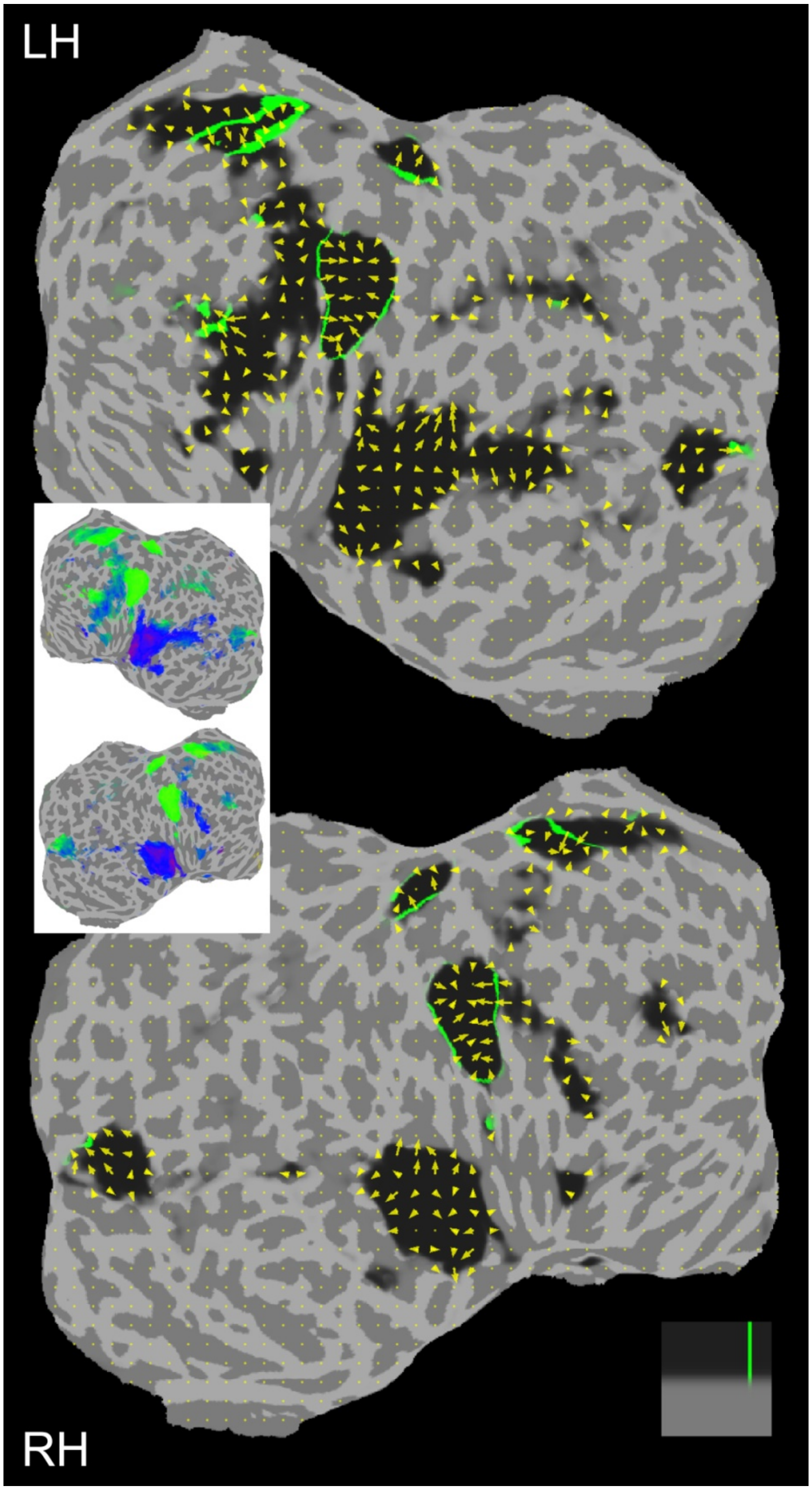

Figure S21. Group-average traveling waves with vector field overlay of the English (L2) to Chinese (L1) listening-memorizing-translating task. Main figure corresponds to Movie S17. Inset corresponds to Figure 5b, right. LH: left hemisphere; RH: right hemisphere.

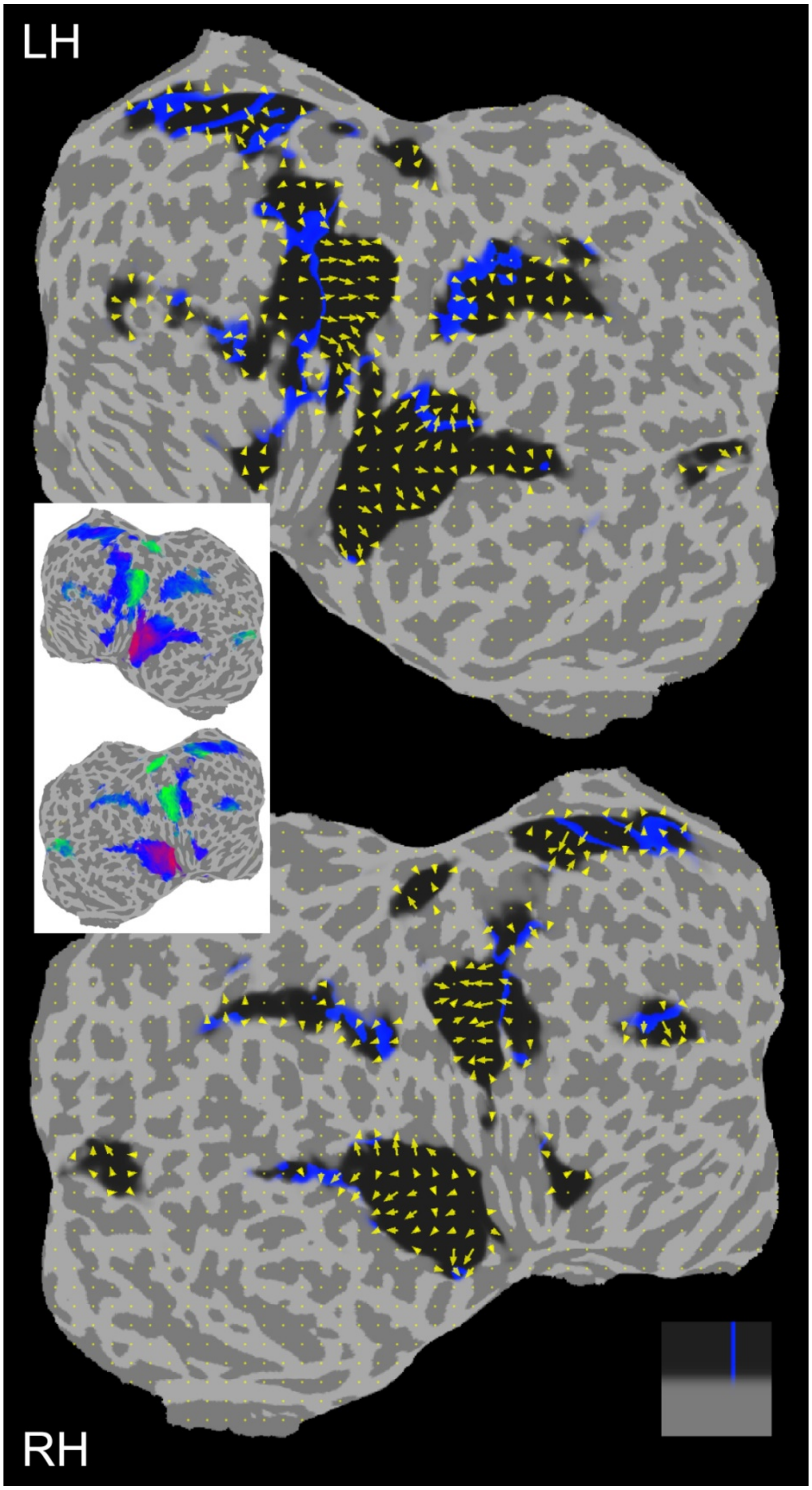

Figure S22. Group-average traveling waves with vector field overlay of the English (L2) to Chinese (L1) listening-memorizing-translating digits task. Main figure corresponds to Movie S18. Inset corresponds to Figure 5c, right. LH: left hemisphere; RH: right hemisphere.

Table S1. Sixteen Chinese (L1) example sentences.

成功的企业投资了这个高级产品
(Successful enterprises invested in this premium product.)
稚气的小学生认真参观了博物馆
(Elementary school students visited this museum.)
联合国成功解决严重的粮食危机
(The United Nation successfully tackled the severe food crises.)
中央政府正在分发防疫知识手册
(The central government is distributing epidemic prevention guidelines.)
印度尼西亚的领导人签署了协议
(Indonesian leaders have signed an agreement.)
植物学专家耐心解释了相关问题
(The botanists patiently explained the relevant issues.)
父母增加了我这一学期的生活费
(My parents have increased my living expenses this semester.)
父母增加了我这一学期的生活费
(Archaeologists stumbled upon prehistoric remains.)
巨大的闪电击中了东方明珠塔顶
(A massive lightning bolt struck the top of Oriental Pearl Tower.)
中国银行聘请了专业的统计人员
(The Bank of China has hired professional statisticians.)
研究生耐心地收集齐了实验数据
(Graduate students were patiently collecting experimental data.)
父亲送给我一个包装精美的礼物
(My dad gave me a beautifully wrapped gift.)
新开发的药物降低了疾病死亡率
(This newly developed drug has reduced the mortality rate of the disease.)
动物园员工正在喂养一头小狮子
(The zoo staff is feeding a lion cub.)
出入境工作人员仔细检查身份证
(The immigration officers carefully check all identification cards.)
调皮的孩子捕捉到了许多的昆虫
(This naughty child caught many bugs.)

Table S2. Sixteen English (L2) example sentences.

Staff announced the arrival of the train at the airport station.
We are anticipating the result of our campaigning.
They are assessing the impact of this on the environment.
Winter attracted more than a million visitors to the city.
A top criminal psychologist cast doubt on the theory.
The government will cease restrictions at the end of this year.
Women comprise majority of hospital medical staff.
He was dealing cards in an illegal gambling operation.
This foundation has funded a great many financial projects.
The principal fulfilled his promise of announcing a date.
The main body will extend to around twelve feet naturally.
Some analysts have estimated its current popularity.
We had already established contact with the museum.
The groups emphasize the need for a good physical approach.
I will be discussing the situation with colleagues tomorrow.
Thousands of soldiers are distributing food to the refugees.

Table S3. Laterality index estimated from L1 and L2 maps (Figure 4). LH: Counts of vertices in the left hemisphere that survived a threshold of $P < 0.001$ (uncorrected); RH: Counts of vertices in the right hemisphere that survived a threshold of $P < 0.001$ (uncorrected); RH/LH: The ratio of the vertex counts. The laterality index (LI) was calculated by the standard formula (LH-RH)/(LH+RH) (Seghier, 2008). LI ranged from − 1 (full right dominance) to + 1 (full left dominance). Right hemisphere language laterality was defined as $-1 \leq LI < -0.2$, bilaterality as $-0.2 \leq LI \leq 0.2$, and left hemisphere language laterality as $0.2 < LI \leq 1$. R: reading; L: listening; RA: reading-aloud; S: shadowing; RMR: reading-memorizing-reciting; LMR: listening-memorizing-reciting.

**Task**	**Chinese (L1)**				**English (L2)**			
	**LH**	**RH**	**RH/LH**	**LI**	**LH**	**RH**	**RH/LH**	**LI**
**R**	13588	8945	0.66	0.21	23003	15003	0.65	0.21
**L**	11126	8806	0.79	0.12	11788	8696	0.74	0.15
**RA**	45365	36876	0.81	0.1	68407	62501	0.91	0.05
**S**	38577	32741	0.85	0.08	41608	34402	0.83	0.09
**RMR**	72296	60246	0.83	0.09	87024	79993	0.92	0.04
**LMR**	40105	30360	0.76	0.14	53708	41815	0.78	0.12

Supporting Videos

Movie S1. Traveling waves during a silent reading task in L1.

Movie S2. Traveling waves during a listening task in L1.

Movie S3. Traveling waves during a reading-aloud task in L1.

Movie S4. Traveling waves during a shadowing task in L1.

Movie S5. Traveling waves during a reading-memorizing-reciting task in L1.

Movie S6. Traveling waves during a listening-memorizing-reciting task in L1.

Movie S7. Traveling waves during a silent reading task in L2.

Movie S8. Traveling waves during a listening task in L2.

Movie S9. Traveling waves during a reading-aloud task in L2.

Movie S10. Traveling waves during a shadowing task in L2.

Movie S11. Traveling waves during a reading-memorizing-reciting task in L2.

Movie S12. Traveling waves during a listening-memorizing-reciting task in L2.

Movie S13. Traveling waves during an L1-to-L2 sight interpreting task.

Movie S14. Traveling waves during an L1-to-L2 consecutive speech interpreting task.

Movie S15. Traveling waves during an L1-to-L2 consecutive digit interpreting task.

Movie S16. Traveling waves during an L2-to-L1 sight interpreting task.

Movie S17. Traveling waves during an L2-to-L1 consecutive speech interpreting task.

Movie S18. Traveling waves during an L2-to-L1 consecutive digit interpreting task.

Movie S19. A snapshot of the University of Macau Brain Atlas (UMBA) website.

Movie S20. Tracking 'brainstorms' on an earth-like surface. The group-average activation map of the Chinese reading-memorizing-reciting task (see Figure 4e, left) is displayed on the spherical cortical surface of the left hemisphere of a single subject. Color contours represent the borders of topological areas (Sereno et al., 2022). Moving white strips represent traveling waves.
